# Accuracy of a Smartphone-Based Object Detection Model, PlantVillage Nuru, in Identifying the Foliar Symptoms of the Viral Diseases of Cassava–CMD and CBSD

**DOI:** 10.3389/fpls.2020.590889

**Published:** 2020-12-18

**Authors:** Latifa M. Mrisho, Neema A. Mbilinyi, Mathias Ndalahwa, Amanda M. Ramcharan, Annalyse K. Kehs, Peter C. McCloskey, Harun Murithi, David P. Hughes, James P. Legg

**Affiliations:** ^1^Virus and Vector Ecology Group, International Institute of Tropical Agriculture (IITA), Dar es Salaam, Tanzania; ^2^Department of Molecular Biology and Biotechnology, University of Dar es Salaam, Dar es Salaam, Tanzania; ^3^Bayer Crop Science, Chesterfield, MO, United States; ^4^Department of Entomology, The Pennsylvania State University, University Park, PA, United States; ^5^Agricultural Research Service (ARS) Research Participation Program, Oak Ridge Institute for Science and Education, Oak Ridge, TN, United States; ^6^Department of Biology, The Pennsylvania State University, University Park, PA, United States

**Keywords:** cassava mosaic disease, cassava brown streak disease, e-extension services, Africa, Kenya, Tanzania, image recognition systems, mobile applications for agriculture

## Abstract

Nuru is a deep learning object detection model for diagnosing plant diseases and pests developed as a public good by PlantVillage (Penn State University), FAO, IITA, CIMMYT, and others. It provides a simple, inexpensive and robust means of conducting in-field diagnosis without requiring an internet connection. Diagnostic tools that do not require the internet are critical for rural settings, especially in Africa where internet penetration is very low. An investigation was conducted in East Africa to evaluate the effectiveness of Nuru as a diagnostic tool by comparing the ability of Nuru, cassava experts (researchers trained on cassava pests and diseases), agricultural extension officers and farmers to correctly identify symptoms of cassava mosaic disease (CMD), cassava brown streak disease (CBSD) and the damage caused by cassava green mites (CGM). The diagnosis capability of Nuru and that of the assessed individuals was determined by inspecting cassava plants and by using the cassava symptom recognition assessment tool (CaSRAT) to score images of cassava leaves, based on the symptoms present. Nuru could diagnose symptoms of cassava diseases at a higher accuracy (65% in 2020) than the agricultural extension agents (40–58%) and farmers (18–31%). Nuru’s accuracy in diagnosing cassava disease and pest symptoms, in the field, was enhanced significantly by increasing the number of leaves assessed to six leaves per plant (74–88%). Two weeks of Nuru practical use provided a slight increase in the diagnostic skill of extension workers, suggesting that a longer duration of field experience with Nuru might result in significant improvements. Overall, these findings suggest that Nuru can be an effective tool for in-field diagnosis of cassava diseases and has the potential to be a quick and cost-effective means of disseminating knowledge from researchers to agricultural extension agents and farmers, particularly on the identification of disease symptoms and their management practices.

## Introduction

The steady increase in the world population and changes in climate are adding pressure to agriculture as the need to produce more food intensifies, and pests and diseases exacerbate these threats to food production. Enhancing the management of emerging pests and diseases along with the production of climate-resilient and disease-resistant crops are some of the efforts that are being put in place to prevent the risk of hunger (Fao et al., 2018). Tools and technologies that can be used for early detection and diagnosis of crop diseases and pests are being encouraged to facilitate their management.

Information and Communication Technology (ICT) platforms in the form of the internet, call-centers and SMS have been adopted to disseminate agricultural information to farmers in several regions including Latin America, Asia, and Africa (Qiang et al., 2012; Tsan et al., 2019). These technologies have shown promising results in reducing the knowledge gap between experts and farmers by enabling transfer of information about basic skills, new technologies and production techniques (Furuholt and Matotay, 2011; Qiang et al., 2012; Misaki et al., 2018).

Developing ICT tools that are capable of identifying crop disease and pest damage poses a greater challenge due to the variability of symptoms. However, several novel techniques for disease and pest identification that use image recognition systems have been developed (Barbedo, 2014; Tuhaise et al., 2014; Pethybridge and Nelson, 2015; Prasad et al., 2016; Qin et al., 2016; Sladojevic et al., 2016; Fuentes et al., 2017; Johannes et al., 2017; Ramcharan et al., 2017). The image recognition system developed by Tuhaise et al. (2014) is one of the first systems developed for cassava. It estimates disease severity by evaluating the percentage of root necrosis attributed to cassava brown streak disease (CBSD). Even though this method is effective and might be useful for research purposes it is not practical for farmers as it requires plants to be uprooted.

Most of the image recognition systems developed for diagnosis of plant diseases provide remote, indirect detection where images of diseased plants are uploaded and analyzed prior to sending feedback to the users. Such technologies are useful to researchers but may be less effective for agricultural extension officers and farmers in areas with limited phone networks. PlantVillage Nuru and Leaf Doctor are currently the only publicly available mobile-based applications that can be used for in-field diagnosis of plant diseases (Pethybridge and Nelson, 2015; Ramcharan et al., 2017, 2019). Both applications can be downloaded, free of charge, from the Apple Store and Android PlayStore. However, PlantVillage Nuru is more geared to application in Africa where Android has a very large market share (>85%; StatCounter Global Stats, 2020). PlantVillage Nuru also provides real-time diagnosis and management advice in the absence of a mobile network, making it ideal for use in remote areas.

PlantVillage Nuru was created from a deep learning object detection model that can determine the presence of diseases and pests in plants based on foliar symptoms (Ramcharan et al., 2017, 2019). The model was trained using 2,756 images of cassava leaves with symptoms of cassava pests and diseases, including CBSD, CMD, brown leaf spot (BLS), cassava green mite (CGM), red mite (RM) as well as asymptomatic leaves. Currently Nuru is trained to identify the presence of the most important pest/disease constraints of cassava. These include the viral diseases of cassava (CMD and CBSD) and the damage caused by CGM (sample images are illustrated in Supplementary Material A). In addition, Nuru has been trained to detect damage caused by fall armyworm (FAW), and new extensions will provide identification for maize lethal necrosis disease and early and late blight in potato. All of these will be delivered through one integrated objected detection model that works offline. Together, CMD and CBSD are arguably the greatest causes of economic losses in cassava production in Africa (Legg et al., 2011), while FAW has been reported to cause damage in several major crops including maize, sorghum, rice and sugarcane (Abrahams et al., 2017). These diseases and pests pose a great challenge to food production since cassava and maize are two of the major staple food crops in Africa, South America, and Asia (Pariona, 2019). Moreover, cassava is also used as a source of food for animals and as a raw material for the production of starch (Hillocks et al., 2002). Africa is the largest producer of cassava in the world, accounting for more than 50% of global production (FAOSTAT, 2018), hence management of CMD and CBSD is crucial for the survival of the crop.

Both CMD and CBSD are controlled through the development and deployment of resistant varieties, as well as through the application of phytosanitary measures, including the removal of infected plants during early growth stages and the selection of healthy stems for use as planting material (Dixon et al., 2003; Thresh and Cooter, 2005; Legg et al., 2015; Kawuki et al., 2016). Effective diagnostic methods are vital if these measures are to be successfully implemented. Laboratory methods can be used to test for the presence of the viruses that cause CMD and CBSD (Hong et al., 1993; Monger et al., 2001), however these tests cannot be used at community level, and hence symptom recognition continues to be the most common way of diagnosing CMD, CBSD and CGM-damage (Sseruwagi et al., 2004). Although visual assessment of symptoms is a valuable first-line diagnostic approach for each of these major diseases/pests of cassava, it has not been widely nor effectively applied. Ineffectiveness of the visual assessment of CMD and CBSD is due to the weakly resourced nature of extension systems in most African countries which hinders knowledge transfer from researchers (via extension officers) to farmers, thereby depriving them of relevant information required for accurate diagnosis and management of diseases and pests (Daniel et al., 2013). Mobile-based apps like PlantVillage Nuru have an important potential role to play within extension systems by serving as diagnosis and training tools for extension officers and farmers. In view of the rapid penetration of mobile phone technology in developing countries (GSMA, 2019), such tools will become accessible to the majority of farmers in the near future, which means that their reach will greatly exceed that of existing extension systems.

The mobile-based technologies available for diagnosis of plant diseases are relatively new and have not been around long enough to evaluate their effectiveness in enabling farmers to improve their disease diagnosis capability. The present study evaluates the effectiveness of PlantVillage Nuru for in-field diagnosis of the viral diseases of cassava and compares its accuracy to that of researchers, agricultural extension officers and farmers. Although the primary role of PlantVillage Nuru is for rapid pest/disease diagnosis rather than training, the teaching capability was also evaluated to provide information about the potential of the app to be used as a tool for transferring knowledge on pest and disease management from researchers to agricultural extension officers and farmers.

## Materials and Methods

### PlantVillage Nuru–Cassava Model

The cassava model of PlantVillage Nuru was developed using deep convolutional neural network (CNN) algorithms trained to identify symptoms of CMD and CBSD as well as damage caused by CGM on cassava leaf images (Ramcharan et al., 2017). A total of 2,756 images of asymptomatic and symptomatic cassava leaves from different varieties of cassava plants grown at field sites in coastal Tanzania (Chambezi, Bagamoyo) were selected and annotated by cassava disease experts at the International Institute of Tropical Agriculture (IITA) (Ramcharan et al., 2017). The annotated images were for training the cassava diagnostic model and these included images with symptoms of CBSD (398 images), CMD (388 images), BLS (386 images), CGM (309 images), and RM (415 images) as well as images of asymptomatic leaves (860 images) (Ramcharan et al., 2017). The model was subsequently deployed as a mobile app (PlantVillage Nuru) in Android smartphones and then tested in fields to account for environmental factors and hence determine the best conditions that will enable the model to perform with high accuracy (Ramcharan et al., 2019).

PlantVillage Nuru works by performing real-time analysis of the image displayed on the screen when using the app. The user is directed to point the phone’s camera onto a leaf that does not look healthy and ensure that the image is in focus prior to analysis. Once the image is in focus the user can start the analysis and the app will display boxes indicating the condition it has identified on individual leaflets. Once the user has finished inspecting the whole plant the app provides the user with an overall diagnosis of the condition of the plant followed by advice on management of the disease or pest it has detected. Both the disease diagnosis and advice capabilities of PlantVillage Nuru are available while offline, enabling users to get results even when in remote areas with no network services. PlantVillage Nuru is programmed in multiple languages including English and Swahili; Swahili is widely spoken throughout East Africa as well as in parts of central Africa. On-going efforts are being made to provide access in other languages through a crowd-sourcing translation tool, and a voice command functionality is being integrated into the app to give access to users who are not able to read and write.

PlantVillage Nuru was evaluated by comparing its capability to diagnose cassava diseases to that of expert researchers, other researchers, agricultural extension officers and farmers. This required experts to be highly skilled in determining the presence of cassava diseases based on foliar symptoms. The capability of expert researchers (cassava experts from IITA, Dar es Salaam, Tanzania) to identify foliar symptoms of cassava diseases was therefore determined at the outset.

### Determination of the Capability of Cassava Experts to Diagnose Cassava Diseases Based on Foliar Symptoms

The ability of cassava experts to accurately identify the symptoms of CMD, CBSD and CGM damage, based on symptoms observed from the leaves, was assessed by comparing visual and molecular diagnosis (through conventional PCR or qPCR amplification of the disease-causing viruses). Two cassava experts, each with at least 3 years of work experience on cassava virus diseases, visually diagnosed 75 cassava plants of five different varieties by assessing the presence or absence of symptoms of CMD and CBSD. The experts randomly selected two leaves (one from the top and the other from the bottom part of each cassava plant) and scored the leaves based on the observed condition. The inspected leaves were sampled and used to test for the presence of the disease-causing virus by molecular diagnosis. The accuracy of expert diagnosis was determined by calculating the proportion of plants for which the symptom diagnoses of experts matched those of the virus test results.

### Molecular Identification of the Viruses That Cause CMD and CBSD From Cassava Leaves

Standard protocols for laboratory detection of CMD-causing viruses (Abarshi et al., 2012) and CBSD-causing viruses (Adams et al., 2012) were used. These involved extraction of nucleic acid from the investigated cassava leaves and PCR amplification of the viruses that cause CMD and CBSD. Two leaves, one from the top and the other from the bottom of the cassava plants were sampled from the 75 cassava plants that were investigated. Symptomatic leaflets were labeled, and the leaves were dried prior to analysis.

#### Extraction of Nucleic Acid From Investigated Leaves

Prior the PCR amplification the cassava leaves were dried, ground and total nucleic acid was extracted using a cetyl trimethyl ammonium bromide (CTAB) extraction procedure. Briefly, 1 mL of CTAB buffer (containing 2.0% w/v CTAB, 2.0% PVP, 25 mM EDTA, 2.0 M NaCl, 100 mM Tris HCL pH 8.0, and 0.2% β-mercaptoethanol which was added immediately before extraction) was added to the dried cassava leaves. The leaves were then ground and incubated at 65°C for 15 min to lyse the cells to facilitate the separation of polysaccharides and polyphenols from the cellular material. An equal amount of chloroform:isoamyl alcohol (24:1) was added to the cell lysate to separate the nucleic acids from the cell lysate, the nucleic acid was precipitated from the solution by adding 0.6x volume of cold isopropanol prior to incubation at −20°C for 30 min followed by centrifugation at 13,000 rpm and 4°C for 10 min. The supernatant was discarded and the nucleic acid pellet was washed twice by adding 700 μL of 70% ethanol followed by vortexing and incubating at −20°C prior to centrifugation at 13,000 rpm for 5 min. Afterwards, the ethanol solution was removed, the nucleic acid pellet was air-dried and then resuspended in 100 μL Tris-EDTA buffer (1x). The quantity and quality of the nucleic acid extracts were determined by spectrometry at 260 nm.

#### PCR Amplification of Viruses That Cause CMD

Nucleic acid of the virus that causes CMD in the coastal region (East African cassava mosaic virus – EACMV) was amplified using the primer pair EAB555F and EAB555R, designed to amplify a 560 bp DNA fragment as described by Ndunguru et al. (2005). About 20 ng of total nucleic acid was added to the PCR master mix containing One Taq 2x Master mix with standard buffer (M0482S, New England Biolabs) and 200 nM each of the forward and reverse primers. A negative control (no-template) and positive controls (samples obtained from symptomatic plants maintained in the screen house that had previously been tested and shown to have the virus of interest) were also included in the analysis.

PCR amplification was done using a Veriti thermocycler (Applied Biosystems) with the following cycling conditions: initial DNA denaturation at 94°C for 2 min, followed by 30 cycles of denaturation (at 94°C for 30 s), annealing (at 55°C for 30 s) and extension (at 68°C 40 s), then a final extension at 68°C for 10 min. The PCR products were analyzed by gel electrophoresis using 1% (w/v) agarose gels and 1X TAE buffer. The DNA products were stained with GelRed nucleic acid stain (Biotium, California, United States) and the gels were viewed and photographed using the Syngene GBox system (Syngene, Cambridge, United Kingdom). Samples containing DNA bands of about 560 bp were considered as EACMV positive results.

#### PCR Amplification of Viruses That Cause CBSD

Detection of the viruses that cause CBSD (Cassava brown streak virus and Ugandan cassava brown streak virus) was done by real-time RT-PCR (qPCR) using TaqMan chemistry and primers described by Adams et al. (2012). Four microlitres of the template nucleic acid was added into the PCR reaction mixtures containing 1x PCR buffer, 5.5 mM MgCl_2_, 0.5 mM dNTPs, 300 nM primer, 100 nM probe, 30 nM Rox reference dye, 0.625 Units of Taq DNA polymerase and 0.4 Units of M-MLV- reverse transcriptase into a 25 μL reaction. The Taq DNA polymerase and reverse transcriptase were obtained from Life Technologies (California, United States) while all the reagents in the PCR master mix were obtained from IDT (Iowa, United States). An internal control (Cytochrome oxidase 1), a negative control (no-template), and positive controls (samples obtained from symptomatic plants maintained in screen houses that had previously been tested and shown to have the virus of interest) were also included in the analysis.

The amplification reactions were done using a Stratagene MX3000P qPCR machine (Agilent Technologies, New Jersey, United States) with the following thermo-cycling conditions: 30 min incubation at 48° for reverse transcription, initial denaturation of the cDNA at 95°C for 10 min, 40 cycles of denaturation at 95°C for 15 s and annealing and extension at 60°C for 1 min. Fluorescence data were collected during the 60°C step using Stratagene MxPro Real-time qPCR software version 4 (Agilent Technologies, New Jersey, United States). Based on the amplification curves, samples with cycle threshold (Ct) values below 36 were considered as positive results.

### Evaluation of In-Field Accuracy of PlantVillage Nuru for Diagnosing the Viral Diseases of Cassava Based on Foliar Symptoms

The ability of PlantVillage Nuru to identify symptoms of CMD, CBSD, and CGM damage was tested in the field (using a Huawei P10 smartphone) by selecting 15 plants for each condition, as identified by experts, and five asymptomatic plants, making a total of 50 plants. Of the 15 plants with each disease/pest condition, five plants had intermediate symptoms of the condition, five plants had mild symptoms of the condition and five plants had unclear symptoms of the condition. This last group comprised plants in which symptoms were not typical of symptoms for that condition. Plants in each of these groups were selected by two researchers with at least three years of experience on cassava pests and diseases. For each of the sampled plants, six leaves were assessed, three from the top and three from the bottom part of the plant. During diagnosis with PlantVillage Nuru, the app was pointed at the leaves for a period of 10 s and the symptoms that were detected were identified by boxes that popped up on the diagnosis screen. The degree of congruence between PlantVillage Nuru’s diagnoses and those of the experts, for each of the symptom categories, was determined by calculating the percentage of leaves for which diagnoses matched.

The value of making diagnoses using more than one leaf was determined and the degree to which results from experts and PlantVillage Nuru matched for upper leaves was assessed by comparing results first for leaf 1 (top upper leaf), then for leaves 1 and 2 (two upper leaves), then for leaves 1, 2, and 3 (three upper leaves). The same process was repeated for lower leaves where the one-leaf comparison was done using the first of the lower leaves, the two-leaf comparison using two lower leaves, and the three-leaf comparison using three lower leaves. Finally, for each of the three conditions (CMD, CBSD, and CGM), percentage matches between expert and PlantVillage Nuru were calculated when using between one and six leaves. The one-leaf comparison used the top upper leaf only. Subsequent comparisons used the following sets of leaves: two-leaf comparison (one upper leaf and one lower leaf); three-leaf comparison (two upper leaves and one lower leaf); four-leaf comparison (two upper and two lower leaves), five-leaf comparison (three upper and two lower leaves), and six-leaf comparison (three upper and three lower leaves). Results for all of the conditions were then combined to give an overall matching percentage value between expert and PlantVillage Nuru when using different numbers of leaves for diagnosis. Examples of the leaves that were used for investigating the use of multiple leaves for the diagnosis of the whole plant are illustrated in Supplementary Material B.

In-field and on-screen symptom recognition accuracy of PlantVillage Nuru were compared to determine if there was a difference in the performance when assessing leaves “on-screen” or “in-field.” To do this, pictures were taken of each of the 300 leaves used for the in-field assessment described above (90 leaves from CMD-affected plants, 90 from CBSD-infected plants, 90 from plants with CGM-damage and 30 leaves that were asymptomatic). PlantVillage Nuru was then used to diagnose these pictures on a laptop screen and the accuracy of PlantVillage Nuru in diagnosing each of the four conditions in-field and on-screen was determined by comparing the diagnoses of the AI system with those of the experts.

### Development of the Cassava Symptom Recognition Assessment Tool for Validation of the Cassava Model, on PlantVillage Nuru

The cassava symptom recognition assessment tool (CaSRAT) was developed at IITA based on a scoring matrix for assessing the condition of 170 images that were randomly selected from a local cassava farm in Mkuranga region. These images include cassava leaves that were symptomatic for CMD (30 images), CBSD (69 images), CGM (51 images), and co-infection (8 images) as well as images with symptoms of other conditions (such as mineral deficiency–8 images) and images that could not be properly diagnosed (4 images). The 170 images were reviewed by 10 cassava experts at IITA-Tanzania, with more than 3 years’ experience of working on cassava pests and diseases, and the group made consensus diagnoses for each of the images. These consensus identifications were then used for the image set as a baseline against which to judge the performance of test groups. During the assessment with CaSRAT, the images were presented to individuals being tested using a projector at an interval of 15 s per image and each individual filled in a scoring sheet (Supplementary Material C) with pre-coded scores (i.e., “1” for CMD, “2” for CBSD etc.) indicating the symptoms observed on the cassava leaf images. The scores filled in by the individuals being assessed were then compared to the expert score and the accuracy for symptom recognition for each individual was then calculated as a percentage of leaves that were correctly scored. Detailed information on how the symptom recognition assessment tool was developed can be found in Supplementary Material D.

### Comparison of the Diagnostic Capabilities of Researchers, Extension Officers, Farmers, and PlantVillage Nuru by Using CaSRAT

The diagnostic capabilities of 60 people in three major categories (researchers, extension agents and farmers) were compared. For each major category, there were 10 who had been trained on cassava pests and diseases and 10 who had not. Both sets of researchers were from IITA-Tanzania, whilst the sets of extension officers and farmers (both trained and untrained) were from Mkuranga District, south of Dar es Salaam, Tanzania. The farmers evaluated in this study were all cassava growers and they were from one of the main cassava growing regions in Tanzania. The trained farmers were selected from farmers that had been involved in a study between 2014 and 2016 investigating the effectiveness of community phytosanitation in reducing the effects of cassava diseases (Legg et al., 2017). This group had received training on each of the major pests and diseases of cassava. The untrained farmers were selected from farmers that had not participated in the phytosanitation study. All farmers selected had been growing cassava for at least 5 years. During the assessments of accuracy in symptom recognition for the various groups tested, each individual was shown the CaSRAT images and was requested to score the images based on the symptom/condition that they observed. Average scores were obtained for each set.

To evaluate PlantVillage Nuru using CaSRAT, the app was pointed at a laptop screen showing the CaSRAT images at 15 s intervals. Diagnoses provided by PlantVillage Nuru were used to fill in the CaSRAT scoring sheet for determining the app’s accuracy score by comparing its diagnoses with the consensus diagnoses of the experts. Evaluation of PlantVillage Nuru by CaSRAT was done using four different phones (Huawei P10, Samsung Galaxy 4, Tecno Camon CM and Infinix Hot 5) to determine if there was variation in Nuru’s accuracy based on the type of phone used. Additionally, the CaSRAT assessment was conducted twice in order to evaluate potential improvements over time, firstly using PlantVillage Nuru V1.05 (released in March 2018) and secondly with PlantVillage Nuru V2.6.0-39 (released in June 2020).

### Evaluating the Teaching Capability of the Cassava Model in PlantVillage Nuru

The teaching capability of PlantVillage Nuru was evaluated by determining the ability of agricultural extension agents to identify symptoms of cassava diseases prior to and after using the app. CaSRAT was used to determine the disease diagnosis ability of 30 agricultural extension agents and 50 farmers from Busia County (western Kenya) prior to the introduction and use of PlantVillage Nuru. After the assessment, all the agricultural extension officers and farmers were trained on diseases and pests of cassava and then introduced to the app. Afterwards, both the agricultural extension agents and farmers were divided into two groups, half of whom were given phones with access to PlantVillage Nuru and requested to inspect and collect data from 100 healthy and diseased cassava plants using app, within two weeks. The other half were not given access to PlantVillage Nuru nor asked to collect data from cassava plants. After the two weeks, the symptom recognition ability of extension agents and farmers, with and without PlantVillage Nuru, was assessed using CaSRAT and their symptom recognition accuracy scores were compared.

### Statistical Analysis

Boxplots, single factor ANOVA and Tukey HDS statistics were used to evaluate and compare the accuracy scores obtained from the researchers, agricultural extension agents and farmers. The statistical analyses were done using R Studio Version 1.1.456 (RStudio Inc.). The script used for the statistical analysis is found in Supplementary Material D.

## Results

### Accuracy of Cassava Experts in Diagnosing Symptoms of CMD and CBSD

Comparison of the molecular and visual diagnoses showed that the experts in cassava pests and diseases could determine the presence or absence of either CMD and CBSD in a plant with a high degree of accuracy (95% for CMD and 81% for CBSD), when only one condition is considered (Table 1). The majority of the cases where the experts failed to identify symptoms of diseases were due to the presence of CBSD-causing viruses that produced few or no symptoms, a condition known as latent infection.

**TABLE 1:** Comparison of visual and molecular diagnosis of CMD and CBSD.

Condition	Visual	Molecular	Number of plants	Overall accuracy score	Overall condition
CMD	Present	Present	19	95%	Accurately diagnosed
	Absent	Absent	52		
	Absent	Present	3	5%	Misdiagnosed
	Present	Absent	1		
CBSD	Present	Present	35	81%	Accurately diagnosed
	Absent	Absent	26		
	Absent	Present	9	19%	Misdiagnosed
	Present	Absent	5		

### Ability of PlantVillage Nuru and Cassava Experts to Diagnose Symptoms of the Viral Diseases of Cassava Based on the Examination of Single Leaves

The ability of PlantVillage Nuru to accurately identify symptoms of CMD, CBSD, and CGM-damage, and therefore diagnose the presence of these diseases/conditions, was examined using 90 cassava leaves (from 15 cassava plants) that were either asymptomatic or symptomatic for CMD, CBSD, and CGM-damage. Importantly, although plants were classified by experts as affected by CMD, CBSD, or CGM, not all inspected leaves of those plants expressed symptoms of those diseases/pests. PlantVillage Nuru could identify asymptomatic leaves with an accuracy above 90%, which is higher than the accuracy for symptomatic leaves (21–59%); it could also identify symptoms of CGM-damage (40–56%) and CMD (52–59%) better than CBSD (21% accuracy) (Table 2). Furthermore, PlantVillage Nuru’s accuracy for identification of CBSD symptoms (21%) was significantly lower than that of CMD (59%) and CGM (56%).

**TABLE 2:** Comparison of in-field and on-screen capacity of the PlantVillage Nuru app to identify symptoms of CMD, CBSD, and CGM-damage as well as no symptoms on plants identified by experts as affected by CMD, CBSD, CGM or asymptomatic.

	Level of identification (%)			
Condition of the plants	Nuru in-field	Nuru on-screen	Expert on-screen	Expert-Nuru agreement
CMD	59%	52%	100%	52%
CBSD	21%	21%	74%	44%
CGM-damage	56%	40%	76%	51%
Asymptomatic	90%	97%	97%	100%
Overall	49%	53%	87%	62%

For all disease/pest damage conditions, mild symptoms were correctly identified by PlantVillage Nuru with much lower accuracy (13–37%) than intermediate symptoms (27–83%), whilst symptoms that were considered to be unclear were identified with moderate accuracy (23–63%) (Table 3). Examples of leaf images showing intermediate, mild and unclear symptoms are illustrated in Supplementary Material A. Plants with “intermediate” symptoms had more distinctive and uniformly distributed patterns of disease expression than those with mild symptoms, where symptoms were less prominent on a smaller proportion of leaves. Plants with “unclear” symptoms had symptoms that could be confused with other diseases or conditions, such as mineral deficiency, or brown leaf spot.

**TABLE 3:** Capability of the cassava AI Nuru app to identify symptoms of cassava pests and diseases in the leaves of plants identified by experts as affected by CMD, CBSD, CGM based on the severity of the symptoms.

Condition	Severity	No. of leaves	No. of leaves accurately diagnosed	% Accuracy of symptom recognition
CMD	Intermediate	30	25	83%
	Mild	30	11	37%
	Unclear	30	17	57%
CBSD	Intermediate	30	8	27%
	Mild	30	4	13%
	Unclear	30	7	23%
CGM	Intermediate	30	24	80%
	Mild	30	7	23%
	Unclear	30	19	63%

The on-screen accuracy of PlantVillage Nuru for identifying symptoms of CMD, CBSD, and CGM-damage as well as asymptomatic leaves was similar to the in-field accuracy (Table 2). PlantVillage Nuru’s accuracy for identifying symptoms of CMD and CGM-damage in-field (59 and 56%, respectively) was slightly higher for than that obtained from on-screen diagnosis in CaSRAT (52 and 40%, respectively) while accuracy for identifying CBSD symptoms was the same in both cases (21%). On the other hand, the on-screen accuracy of PlantVillage Nuru for identification of asymptomatic leaves (97%) was slightly higher than that of in-field diagnosis (90%). Overall, accuracy scores for PlantVillage Nuru “in-field” and “on-screen” were very similar (Table 2), which demonstrates the validity of using CaSRAT to compare the “on-screen” accuracy of PlantVillage Nuru with that of humans.

The ability of the experts to diagnose CBSD and CGM-damage on-screen (Table 2), using the images obtained from the 300 leaves (of 50 plants: 15 CMD, 15 CBSD, 15 CGM, 5 asymptomatic), was less than for the in-field analysis of the 50 whole plants. Although humans correctly identified CMD symptoms from 100% of the images obtained from plants tagged in the field as affected by CMD and 97% of images were correctly identified as asymptomatic, levels of image recognition were lower for CBSD (74%) and CGM (76%). The reason for this is that several of the leaves from plants tagged as affected by CBSD or CGM did not express symptoms of these conditions. CBSD-infected plants typically express weak symptoms or no symptoms at all on upper leaves whilst CGM symptoms are only prominent on upper leaves. For this reason, the use of multiple leaves for diagnosis of the whole plant was hypothesized as a means to improve the diagnostic accuracy of PlantVillage Nuru since cassava experts usually inspect the whole plant instead of a few leaves.

**FIGURE 1:**
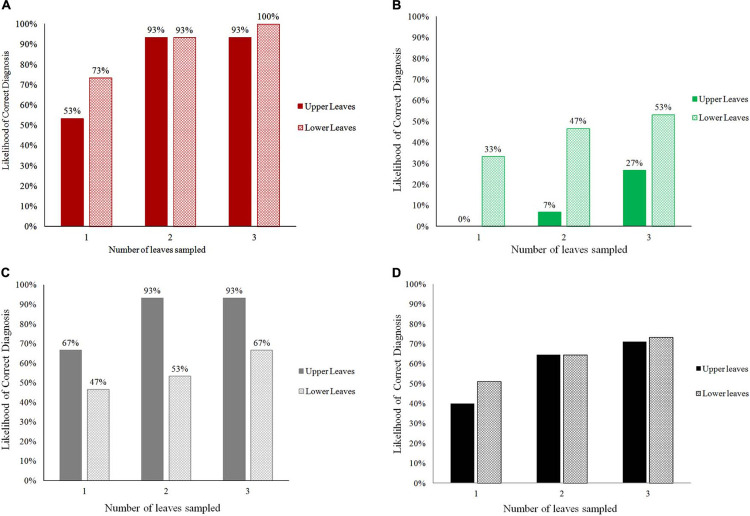
The overall likelihood of obtaining correct diagnoses for CMD **(A)**, CBSD **(B)**, CGM-damage **(C)**, and all conditions **(D)** when using the cassava AI Nuru based on the number and position (upper or lower) of leaves sampled from 90 cassava plants.

**FIGURE 2:**
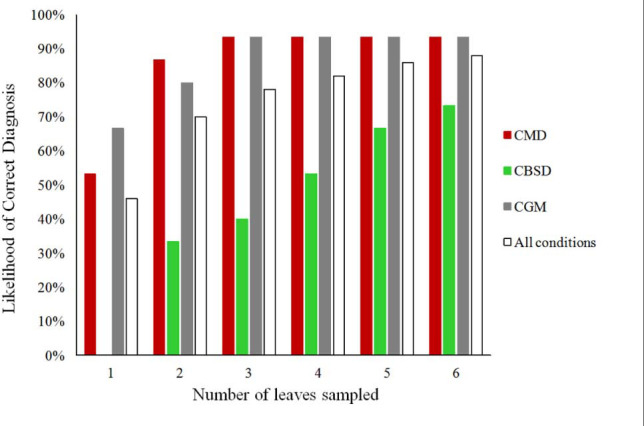
The likelihood of obtaining a correct diagnosis for CMD, CBSD, CGM-damage, and all the three conditions from cassava leaves obtained from 90 plants by using the PlantVillage Nuru app for symptoms based on the number of leaves sampled.

When the number of leaves examined by PlantVillage Nuru was increased, the diagnosis capacity for all three conditions (CMD, CBSD, and CGM) improved greatly (Figure 1). The use of two leaves per plant improved PlantVillage Nuru’s diagnosis capacity for CMD from 53 to 93% for upper leaves and from 73 to 93% for lower leaves, CGM-damage from 67 to 93% for upper leaves and from 47 to 53% for lower leaves, while at least three leaves were required to achieve a similar level of improvement for diagnosis of CBSD (from 0 to 27% for upper leaves and 33 to 53% for lower leaves) (Figure 2).

The location of leaves had an impact on PlantVillage Nuru’s accuracy for identifying symptoms of CGM-damage and CBSD but not CMD. CGM-damage was more accurately identified using upper leaves while CBSD symptoms were more accurately identified using lower leaves. However, the use of six leaves (3-upper and 3-lower) provided the highest likelihood of diagnosis of all conditions, at which the diagnosis of CMD, CBSD and CGM-damage were diagnosed with 93, 73 and 93% accuracy (Figure 1). For this reason, the use of three upper and three lower leaves was the recommended number of leaves suggested for improving the accuracy of the cassava model in Nuru. This is the model that is now implemented in the currently available PlantVillage Nuru app in the Android PlayStore.

### Ability of PlantVillage Nuru, Agricultural Extension Agents and Farmers to Diagnose Symptoms of CMD, CBSD, and CGM on Cassava Leaves

**FIGURE 3:**
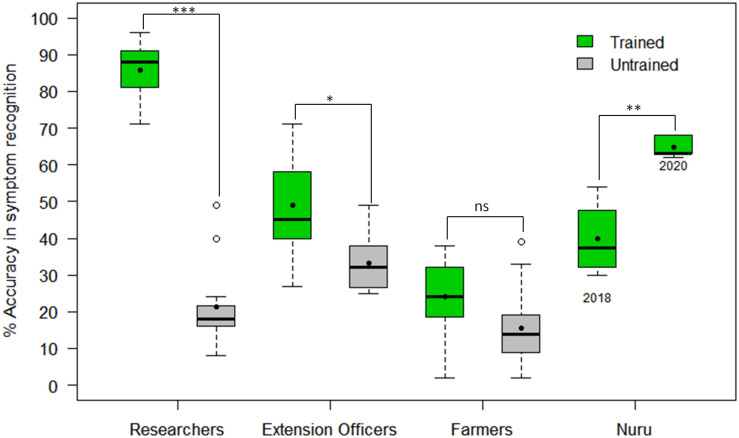
Comparison of accuracy in recognizing symptoms of CMD, CBSD and CGM damage by PlantVillage Nuru, researchers, agricultural extension officers and farmers who have and those who have not previously received training on cassava diseases and pests (*n* = 22 for each paired comparison). The black line within the boxes represents the median, the top and bottom of the box represent the 75th and the 25th percentiles, the whiskers represent the maximum and minimum values while the symbols (●) and (○) represent the mean and the outlier.

The CaSRAT was used to determine the ability of individuals and groups to accurately diagnose foliar symptoms. The researchers trained on cassava pests and diseases were able to identify symptoms of CMD, CBSD, and CGM-damage with higher accuracy than untrained researchers, agricultural extension officers and farmers (Figure 3). The mean accuracy score of symptom recognition by trained researchers (86%) was about four times higher than that of untrained researchers (21%) (Welch two-sample *t*-test *p* = 4.30E−11), almost twice as high as that of trained agricultural extension officers (49%) (Tukey HSD *p* < 0.001) and three times higher than that of trained farmers (23%) (Tukey HSD *p* < 0.001). However, there were small differences between the mean accuracy scores of trained and untrained agricultural extension officers (49 and 32%, respectively, Tukey HSD *p* < 0.05) and trained and untrained farmers (23 and 12%, respectively, Tukey HSD *p* > 0.5).

The majority of images that were misdiagnosed by farmers, trained and untrained, had symptoms of CBSD and CGM-damage; these accounted for 37–42% of all misdiagnoses (Supplementary Material E1). The untrained farmers misdiagnosed leaves with CBSD symptoms as healthy, i.e., asymptomatic (24% of all misdiagnoses), and CGM-damage as CBSD and healthy (14% of all misdiagnoses for each condition). On the other hand, trained farmers most commonly confused symptoms of CBSD with those of CGM-damage (12% of all misdiagnoses) and symptoms of CGM-damage with those of CBSD (20% of all misdiagnoses).

Similarly, the majority of misdiagnoses by agricultural extension officers, trained and untrained, were also due to symptoms of CBSD (30%) and CGM-damage (45%) (Supplementary Material E2). Untrained agricultural extension officers confused symptoms of CBSD with those of CMD (22% of all misdiagnoses) and symptoms of CGM-damage with those of CBSD (23% of all misdiagnoses). Trained agricultural officers also confused symptoms of CGM-damage with those of CBSD (28% of all misdiagnoses), however, they seemed to be less clear with symptoms of CBSD as they misdiagnosed some of the leaves with CBSD symptoms as CMD-infected (11% of all misdiagnoses) and CGM-damage (10% of all misdiagnoses).

Untrained researchers also confused symptoms of CBSD (41% of misdiagnoses) and CGM-damage (39%) more than CMD, while the majority of the misdiagnoses obtained from trained researchers were due to CGM-damage and co-infection (34 and 37% of all misdiagnoses; Supplementary Material E3). The untrained researchers seemed to have confused symptoms of CBSD with those of CMD (11% of all misdiagnoses), CGM-damage (10% of all misdiagnoses) as well as healthy (7% of all misdiagnoses) and other conditions (14% of all misdiagnoses). On the other hand, trained researchers confused symptoms of CGM-damage with those of CMD and CBSD (12% of all misdiagnoses for each condition).

Most of the misdiagnoses due to symptoms of co-infection were obtained from leaves that had symptoms of both, CBSD and CGM-damage (Supplementary Material E), and most of these were identified by researchers and agricultural extension officers to have either CBSD or CGM only. However, the number of images with symptoms of co-infection were few hence more images are required for a proper analysis.

Nuru was able to identify disease symptoms with 54% accuracy when tested on the 170 images used with the CaSRAT, using the same phone that was used for the in-field assessment. However, Nuru’s accuracy for symptom identification was lower (30–41%) for the other three phones assessed using the cassava model V1.05 (Nuru, 2018) (Table 4). Although the average accuracy score of Nuru, 2018 with four phones (40 ± 10%) was slightly lower than that of trained agricultural extension officers (49%) (Tukey HSD *p* > 0.5), its score in 2020 (65 ± 3%) was significantly higher (Tukey HSD *p* < 0.05). The accuracy of the cassava model V.2.6 in Nuru, 2020 was higher for all four different phones that were evaluated (62–68% overall accuracy score) suggesting that PlantVillage Nuru’s improvements also enabled the app to perform better in different phones.

**TABLE 4:** Comparison of overall accuracy of Nuru for identification of foliar symptoms of cassava mosaic disease (CMD), cassava brown streak disease (CBSD), and damage caused by cassava green mites (CGM) on cassava leaves based on the cassava symptom recognition assessment tool (CaSRAT).

Year	March 2018				June 2020				
Phone type	Samsung Galaxy 4	Infinix Hot 5	Tecno Camon CM	Huawei P1O	Samsung Galaxy A10	Infinix Hot 7	Tecno Camon CM	Tecno Spark3 Pro	Huawei P30
Overall accuracy score	30%	41%	34%	54%	62%	63%	68%	63%	68%
Average accuracy score	40 ± 10%				65 ± 3%				

### The Teaching Capability of Nuru

**FIGURE 4:**
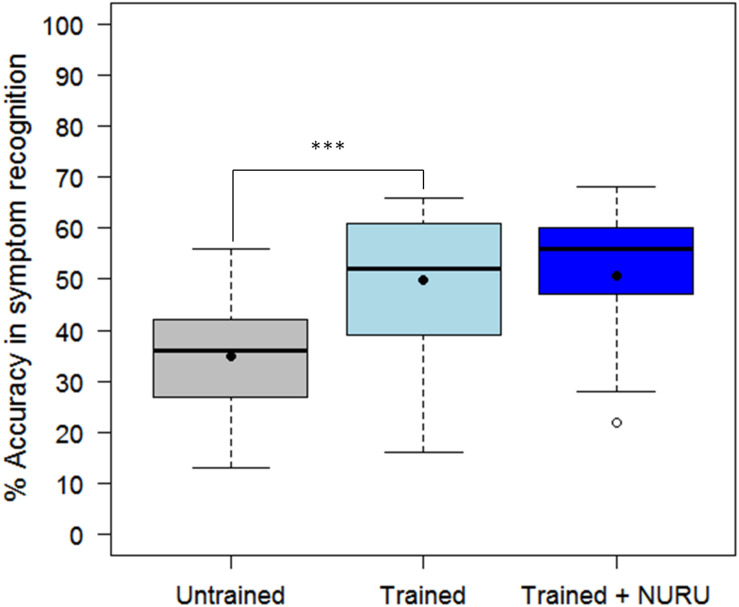
Comparison of the ability of 30 agricultural extension officers and 50 farmers to accurately recognize symptoms of CMD, CBSD, and CGM-damage before and after receiving training on cassava pests and diseases. The black line within the boxes represents the median, the top and bottom of the box represent the 75th and the 25th percentiles, the whiskers represent the maximum and minimum values while the symbols (●) and (○) represent the mean and the outlier.

**FIGURE 5:**
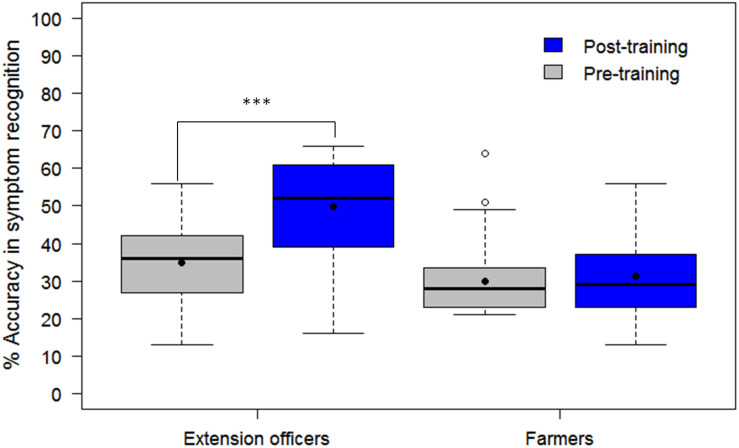
Comparison of the ability of 30 agricultural extension officers to accurately recognize symptoms of CMD, CBSD, and CGM-damage before and after receiving training on cassava pests and diseases as well as after using the PlantVillage Nuru app for diagnosing cassava diseases. The black line within the boxes represents the median, the top and bottom of the box represent the 75th and the 25th percentile, the whiskers represent the maximum and minimum values while the symbols (●) and (○) represent the mean and the outlier.

The assessment tool (CaSRAT) was also used to evaluate the teaching capability of Nuru by determining the ability of agricultural extension officers and farmers trained on cassava pests and diseases to correctly identify symptoms of diseases after using PlantVillage Nuru for about two weeks. There was a significant increase in the ability of the extension agents to identify symptoms of CMD, CBSD, and CGM after the training as evidenced by the increase in their mean accuracy score from 34.9 ± 10.9 to 49.9 ± 13.5% (Tukey HSD *p* < 0.005) (Figure 4). However, this was not the case for the farmers where there was only a marginal change in their accuracy score after training, from 29.9 ± 9.7 to 31.2 ± 10.2% (Tukey HSD *p* > 0.5). There was no significant difference in the mean accuracy scores of the agricultural extension officers before and after using PlantVillage Nuru, from 49.9 ± 13.5 to 50.5 ± 14.5% (Tukey HSD *p* > 0.5). However, the range of the accuracy scores obtained after training and Nuru usage (46–60%) was slightly narrower than that obtained from agricultural extension officers who had only received training without using Nuru (38–61%) (Figure 5).

The extension officers in this group also seemed to confuse symptoms of CBSD with those of CMD (14–19% of all misdiagnoses) and CGM-damage (19–27% of all misdiagnoses) as well as symptoms of CGM-damage with those of CMD (8–12% of all misdiagnoses) and CBSD (16– 20% of all misdiagnoses) (Supplementary Material E5). The percent of images that were misdiagnoses did not differ much after training and the use of PlantVillage Nuru for two weeks.

## Discussion

Development of smartphone-based technologies for diagnosis of disease and pest damage on plants requires input from experts who understand the phenotypes of the diseases and pests. Therefore, it is important to evaluate the expertise of these experts to ensure that the information used to develop such technologies is as accurate as possible. Quantifying the knowledge of the experts also provides a baseline that can be used to evaluate the effectiveness of the developed technologies. The present study examined the expertise of the experts who generated datasets used for the development of an object-detection model for cassava disease diagnosis based on foliar symptoms, known as PlantVillage Nuru. The symptom recognition capability of PlantVillage Nuru was compared to that of the experts and its intended users so as to determine the effectiveness of the app. Since PlantVillage Nuru was developed as a diagnosis and training tool, its capacity to train users was also evaluated to determine if the app can be used to train its users to recognize the symptoms of diseases and pests affecting their plants and hence improve their diagnosis capability.

### Accuracy of Cassava Experts in Diagnosing Symptoms of CMD and CBSD

Cassava experts were able to achieve a high level of accuracy in correctly identifying the disease affecting cassava plants (CMD or CBSD) using visual-based symptom recognition on leaves which were confirmed to be healthy or contain the disease-causing viruses by molecular diagnostic methods. This may represent the first time that such a visual inspection vs. molecular diagnostics comparison has been made for CMD, and it confirms the otherwise widely held view that CMD is relatively easy to identify based on visual symptom assessments (Abarshi et al., 2012). The small number of false negatives associated with CMD infection, identified in the present study, were thought to be due to latent infection that had not resulted in disease symptom expression.

CBSD is known to have more cryptic symptoms than CMD (Nichols, 1950) and more frequent latent infection (Adams et al., 2012). Furthermore, the expression of foliar symptoms in CBSD-infected plants has been reported to vary between leaves on the plants, cassava variety, growing conditions (temperature, rainfall, and altitude), age of the plant and the virus isolate involved in causing the disease symptoms (Hillocks and Jennings, 2003; Mohammed et al., 2012; Shirima et al., 2019, 2020). It is therefore unsurprising that there was a lower level of congruence between symptom vs. virus testing identifications for CBSD than was observed for CMD.

However, the overall high level of accuracy in the visual assessment of CMD and CBSD infection achieved by the experts provided a strong basis both for the previous development of the PlantVillage Nuru app (Ramcharan et al., 2019), as well as the use of expert diagnoses as a benchmark for comparison of diagnoses made by other groups as well as the PlantVillage Nuru app itself.

### Comparison of the Ability of PlantVillage Nuru and Cassava Experts in Diagnosing Symptoms of the Viral Diseases of Cassava Based on Examination of One or More Leaves

When PlantVillage Nuru was used to identify the symptoms of CMD, CBSD, and CGM-damage (using single leaves), it was partially accurate for CMD (58% accuracy score) and CGM (56% accuracy score), but mostly inaccurate for CBSD (21% accuracy score). This highlights the fact that single-leaf diagnoses using a smartphone app such as PlantVillage Nuru are unreliable, partly due to the difference in the severity of the symptoms PlantVillage Nuru can recognize, but also resulting from the uneven distribution of symptoms within cassava plants, particularly for CBSD (Nichols, 1950).

The distinction of sampled plants into those with “mild,” “intermediate,” and “unclear” symptoms gave rise to large differences in the accuracy of single leaf diagnoses by PlantVillage Nuru. The accuracy for diagnosis of intermediate symptoms of CMD and CGM-damage was about 20% higher than that of unclear symptoms and the overall diagnosis accuracy. The diagnostic capacity of Nuru for leaves with mild symptoms was low for all the three conditions (CMD, CBSD, and CGM-damage), probably because images selected for training the app were mostly of leaves with clear symptoms. The overall lower accuracy in identifying CBSD symptoms has been reported previously for PlantVillage Nuru (Ramcharan et al., 2019), and it is a widely published fact that CBSD symptoms are cryptic, seasonally variable and can be difficult to identify, even for experienced researchers (Nichols, 1950; Hillocks and Thresh, 2000).

All of the pest and disease conditions of cassava (especially CMD, CBSD, and CGM) have patterns of symptom expression that vary greatly within plants, from plant to plant, and between varieties and they are affected also by virus strain variation, weather conditions and other environmental factors (Hahn et al., 1980; Thresh et al., 1994; Owor et al., 2004a, b; Mohammed et al., 2012). A plant with a recent vector-borne infection of CMD only expresses symptoms in upper leaves (Sseruwagi et al., 2004) and CGM damage is always most prominent on upper leaves (Onzo et al., 2005) while CBSD symptoms are usually present on leaves toward the bottom of the plant (Nichols, 1950). By definition, asymptomatic plants are uniformly symptom-free, which explains the much higher level of accuracy (90%) achieved by PlantVillage Nuru in identifying this condition from a single leaf. These factors highlight the importance of applying multi-leaf assessments when using PlantVillage Nuru. The same principle would also apply to any other app attempting to deliver phone-based diagnoses of cassava diseases and pest damage.

The use of two leaves (one upper and one lower) improved PlantVillage Nuru’s diagnostic capability for CMD and CGM-damage to a similar accuracy to that of the cassava experts, however, this was not the case for CBSD which required six leaves to approach the expert’s accuracy (accuracy score > 74%). Hence the use of six leaves was adopted as the means for diagnosis of the whole plant as it provided PlantVillage Nuru with a better chance of identifying disease symptoms and therefore improved its performance, even when the severity of the symptoms varied. The use of six leaves mimics the approach that a researcher would take in the field, in which both upper and lower parts of the plants would be inspected in order to confirm the presence of CMD, CBSD or CGM symptoms (Hillocks and Thresh, 2000; Sseruwagi et al., 2004).

### Comparison of the Ability of PlantVillage Nuru, Agricultural Extension Agents and Farmers to Correctly Diagnose Symptoms of CMD, CBSD, and CGM on Cassava Leaves

We compared the diagnostic capability of PlantVillage Nuru (for CMD, CBSD and CGM-damage) to that of its intended users—agricultural extension officers and farmers—to assess the potential beneficial effects of PlantVillage Nuru for these groups. PlantVillage Nuru had the same overall accuracy for identifying symptoms of CMD, CBSD, and CGM-damage as trained agricultural extension officers, who could identify disease symptoms better than untrained researchers, untrained agricultural extension officers and trained or untrained farmers.

The large difference in the symptom identification ability of the trained researchers compared to untrained researchers highlights the difficulty of diagnosing symptoms of CMD, CBSD, and CGM-damage such that only well-trained individuals can do so effectively. By contrast, the difference between the symptom recognition accuracy scores of the trained farmers and agricultural extension officers suggested that the vertical transfer of knowledge from researchers to extension agents is currently inefficient. In order to address this problem, a rigorous training programme might be needed to improve the ability of the agricultural extension agent and farmers to identify and differentiate symptoms of the diseases and conditions that they encounter, and efforts should be made to improve the efficiency with which agricultural extension information flows down to farmers. In order to achieve this, it will also be necessary to tackle the resource constraints which are common features of agricultural knowledge transfer systems in sub-Saharan Africa (Azumah et al., 2018). The ability of PlantVillage Nuru to diagnose disease symptoms with a similar level of accuracy to that achieved by experts, when using multiple leaves, indicated that the app might be able fill the knowledge gap between researchers and agricultural extension officers as well as farmers. Digital tools such as PlantVillage Nuru offer great potential for extending the reach and improving the efficiency of agricultural knowledge transfer systems in sub-Saharan Africa, as telecommunications networks continue their rapid expansion through the continent.

### Evaluation of the Teaching Capability of PlantVillage Nuru

We demonstrated that training alone delivered significant improvements in accuracy of disease diagnosis for extension officers but not farmers, indicating that the applied training method might not have been suitable for farmers. Hence further investigations are recommended to determine the most suitable methods for training farmers and how to evaluate the effectiveness of such trainings. Training with PlantVillage Nuru resulted in a slight improvement in the symptom recognition capacity of both agricultural extension officers and farmers, suggesting that a longer period of time might be required to observe more substantial changes in the symptom recognition capacity of its users.

The training potential of PlantVillage Nuru is based on its ability to show users the symptoms of the diseases/conditions present in the leaves as it is being used (as illustrated in the Supplementary Videos for Healthy-diagnosis, CMD-diagnosis, CBSD-diagnosis, and CGM-diagnosis). This helps users to become familiar with the characteristic symptoms of each of the disease/pest damage types, which over time should enable them to recognize each of these conditions without the aid of PlantVillage Nuru. This learning function is reinforced by PlantVillage Nuru’s library containing images of disease symptoms that the user can access and learn from, in the absence of a mobile network. Furthermore, if the user has access to a mobile network, PlantVillage Nuru can connect the users to researcher experts who can assist in diagnosis of the condition of the plant, through a platform where the user is able to ask questions and share images of their plants (as seen at https://plantvillage.psu.edu/posts). Furthermore, PlantVillage Nuru can link users with the PlantVillage database where users can get information on agricultural practices, diseases, and pests as well as their management techniques for different crops.

Hence, with time, PlantVillage Nuru can provide a quick, cost-effective and easily accessible means for disseminating knowledge and ensuring continuous training of agricultural extension agents and farmers, thereby improving their skills in pest/disease identification and management. On-going work in western Kenya indicates that Nuru is improving disease diagnosis skills of cassava farmers suggesting that efforts to scale out the use of Nuru across the cassava-growing regions of Africa will improve farmers’ recognition and knowledge of cassava diseases/pests, which will contribute to improved disease/pest control and greater productivity.

### Improvements Made on PlantVillage Nuru

The model that we tested here has already been updated and continuous improvements are being made as more data become available, so the diagnosis capacity of PlantVillage Nuru is expected to increase over time. Improvements in smartphone technology will also contribute to better performance of the app, since newer smartphone models tend to have better cameras which enhance sensitivity and accuracy of PlantVillage Nuru.

The diagnostic models used to develop Nuru are publicly available for independent validation, which allows their reliability to be assessed in diverse geographic regions where different varieties of the investigated plants are grown. This may enable modification of the disease diagnostic models to improve performance based on the condition and variety of the host plant as well as identification of conditions that may need to be included in the training datasets. “Open science” models from other sectors, like genome biology and genetic diseases, have led to rapid advances in the application of machine learning approaches to the development of diagnostics.

Additional symptom types that have been proposed for inclusion within the cassava PlantVillage Nuru model include nutrient-deficiency and fungal infection (Howeler, 2002; Hillocks et al., 2002), although these are currently of much lower importance in sub-Saharan Africa than the cassava viruses and CGM. However, the accuracy of the model might be reduced by increasing the number of different conditions with similar symptoms. Therefore other tools which could provide complementary simple, cheap and rapid means for in-field diagnosis of plant diseases (such as pathogen-based biosensors) could be used to enhance the capability of PlantVillage Nuru. Pathogen-based biosensors use the pathogen’s antibodies or nucleic acid for diagnosis (Khater et al., 2017) and hence could act as a confirmatory test for cases where disease symptoms cannot be determined or if there is latent infection. Since pathogen-based biosensors have not yet been developed for in-field diagnosis of the viral diseases of cassava, symptom recognition and lab-based assays will continue to remain the main methods for diagnosis of CMD and CBSD in the immediate future. Therefore, Nuru has an important role to play in building disease diagnostic capacity of agricultural extension officers and farmers.

## Conclusion

The present study has shown that PlantVillage Nuru can be as effective as experts in identifying the symptoms of the viral diseases of cassava (CMD and CBSD) and CGM-damage. PlantVillage Nuru gave a high level of symptom recognition accuracy, which was better than that achieved by agricultural extension officers and farmers, suggesting that it can be used for increasing their diagnostic capacity for the viral diseases of cassava as well as CGM. Since the disease diagnostic models in PlantVillage Nuru are continuously being improved and more knowledge is added as it becomes available, the app provides agricultural extension officers and farmers with an ever-improving direct link to experts and expert knowledge. These features give PlantVillage Nuru and mobile-based apps that can effectively diagnose symptoms of disease and pest damage the ability to revolutionize disease and pest management in agriculture.

The rapid penetration of affordable smartphone technology throughout rural Africa will certainly ensure that the platform for widespread access to such apps will be in place within the near future. Raising awareness amongst farming communities about the availability and utility of apps such as PlantVillage Nuru is an important next step in promoting this process and will require determined and innovative efforts from stakeholders in agricultural development supported by teams of IT specialists. Crucially, however, the success of these endeavours will ultimately depend on farmers’ access to affordable control measures. Although some of this, such as advice on cultural control techniques, can be delivered through apps, elements such as the provision of high-quality seed/planting material of pest/disease resistant varieties will require investment in more traditional on-the-ground extension approaches. As governments increasingly seek to promote the application of ICT solutions to agricultural development in Africa, strong parallel efforts will be required to strengthen variety development, deployment and dissemination systems as well as sustainable approaches for the delivery of other inputs required for effective pest and disease management.

## Data Availability Statement

The raw data supporting the conclusions of this article will be made available by the authors, without undue reservation, to any qualified researcher.

## Ethics Statement

Ethical review and approval was not required for the study on human participants in accordance with the local legislation and institutional requirements. The patients/participants provided their written informed consent to participate in this study.

## Author Contributions

LM, NM, MN, AR, AK, HM, PM, and JL collected the data. LM and JL prepared the manuscript. JL and DH supervised the projects. All authors contributed to the article and approved the submitted version.

## Conflict of Interest

The authors declare that the research was conducted in the absence of any commercial or financial relationships that could be construed as a potential conflict of interest.

## References

[B1] Abarshi M. Mohammed I. Jeremiah S. Legg J. Kumar L. Hillocks R. (2012). Multiplex RT-PCR assays for the simultaneous detection of both RNA and DNA viruses infecting cassava and the common occurrence of mixed infections by two cassava brown streak viruses in East Africa. *J. Virol. Methods* 179 176–184. 10.1016/j.jviromet.2011.10.020 22080852

[B2] Abrahams P. Beale T. Cock M. Corniani N. Day R. Godwin J. (2017). *Fall Armyworm Status: Impacts and Control Options in Africa: Preliminary Evidence Note.* Oxfordshire: Centre for Agriculture and Biosciences International.

[B3] Adams P. Abidrabo P. Miano W. Alicai T. Kinyua M. Clarke J. (2012). High throughput real-time RT-PCR assays for specific detection of cassava brown streak disease causal viruses and their application to testing planting material. *Plant Pathol.* 62 233–242. 10.1111/j.1365-3059.2012.02622.x

[B4] Azumah B. Donkoh S. Awuni A. (2018). The perceived effectiveness of agricultural technology transfer methods: evidence from rice farmers in Northern Ghana. *Cogent Food Agric.* 4:1 10.1080/23311932.2018.1503798

[B5] Barbedo J. (2014). An automatic method to detect and measure leaf disease symptoms using digital image processing. *Plant Dis.* 98 1709–1716. 10.1094/PDIS-03-14-0290-RE 30703885

[B6] Daniel E. Bastiaans L. Rodenburg J. Center A. R. Schut M. Mohamed J. K. (2013). *Assessment of Agricultural Extension Services in Tanzania. A Case Study of Kyela, Songea Rural, and Morogoro Rural Districts. Internship Report in Plant Sciences. Crops Systems Analysis Group.* Wageningen: University and Research Centers.

[B7] Dixon A. Bandyopadhyay R. Coyne D. Ferguson M. Ferris R. Hanna R. (2003). Cassava: from poor farmers’ crop to pacesetter of African rural development. *Chron. Horticult.* 43 8–15.

[B8] Fao I. F. A. D. Unicef W. F. P., and Who. (2018). *The State of Food Security and Nutrition in the World. Building Climate Resilience for Food Security and Nutrition.* Rome: FAO.

[B9] FAOSTAT (2018). *Food and Agriculture Organisation of the United Nations Database.* Paris: FAOSTAT.

[B10] Fuentes A. Yoon S. Kim S. C. Park D. S. (2017). A robust deep-learning-based detector for real-time tomato plant diseases and pests recognition. *Sensors* 17:2022. 10.3390/s17092022 28869539 PMC5620500

[B11] Furuholt B. Matotay E. (2011). The developmental contribution from mobile phones across the agricultural value chain in rural Africa. *Electr. J. Inform. Syst. Dev. Countries* 48 1–16. 10.1002/j.1681-4835.2011.tb00343.x

[B12] GSMA (2019). *The Mobile Economy-Sub-Saharan Africa.* London: Global System for Mobile Communications Association.

[B13] Hahn S. Leuschner K. Ezeilo W. Carpenter A. Khatibu A. Constantin C. (1980). Resistance of Cassava Clones to Cassava Green Mite, *Mononychellus tanajoa*. *Trop. Pest Manag.* 26 265–267. 10.1080/09670878009414410

[B14] Hillocks R. Jennings D. (2003). Cassava brown streak disease: a review of present knowledge and research needs. *Int. J. Pest Manag.* 49 225–234. 10.1080/0967087031000101061

[B15] Hillocks R. Thresh J. (2000). Cassava mosaic and cassava brown streak virus diseases: a comparative guide to symptoms and aetiologies. *Roots* 7 1–8. 10.9734/arrb/2016/26879

[B16] Hillocks R. Thresh J. Bellotti A. (2002). *Cassava: Biology, Production and Utilization.* Wallingford: CABI publishing, 301–318.

[B17] Hong Y. Robinson D. Harrison B. (1993). Nucleotide sequence evidence for the occurrence of three distinct whitefly-transmitted geminiviruses in cassava. *J. Gen. Virol.* 74 2437–2443. 10.1099/0022-1317-74-11-2437 8245859

[B18] Howeler R. (2002). “Cassava mineral nutrition and fertilization,” *Cassava: Biology, Production and Utilization*, eds Hillocks R. J. Thresh J. M. Bellotti A. C. (CAB International), 115–147.

[B19] Johannes A. Picon A. Alvarez-Gila A. Echazarra J. Rodriguez-Vaamonde S. Navajas A. D. (2017). Automatic plant disease diagnosis using mobile capture devices, applied on a wheat use case. *Comp. Electr. Agric.* 138 200–209. 10.1016/j.compag.2017.04.013

[B20] Kawuki R. Kaweesi T. Esuma W. Pariyo A. Kayondo I. Ozimati A. (2016). Eleven years of breeding efforts to combat cassava brown streak disease. *Breed. Sci.* 66 560–571. 10.1270/jsbbs.16005 27795681 PMC5010303

[B21] Khater M. de la Escosura-MuÑiz A. Merkoçi A. (2017). Biosensors for plant pathogen detection. *Biosens. Bioelectron.* 93 72–86. 10.1016/j.bios.2016.09.091 27818053

[B22] Legg J. Jeremiah S. Obiero H. Maruthi M. Ndyetabula I. Okao-Okuja G. (2011). Comparing the regional epidemiology of the cassava mosaic and cassava brown streak pandemics in Africa. *Virus Res.* 159 161–170. 10.1016/j.virusres.2011.04.018 21549776

[B23] Legg J. Lava P. Makeshkumar T. Ferguson M. Kanju E. Ntawuruhunga P. (2015). Cassava virus diseases: biology, epidemiology and management. *Adv. Virus Res.* 91 85–142. 10.1016/bs.aivir.2014.10.001 25591878

[B24] Legg J. Ndalahwa M. Yabeja J. Ndyetabula I. Bouwmeester H. Shirima R. (2017). Community phytosanitation to manage cassava brown streak disease. *Virus Res.* 241, 236–253. 10.1016/j.virusres.2017.04.020 28487059 PMC5669585

[B25] Misaki E. Apiola M. Gaiani S. Tedre M. (2018). Challenges facing sub-Saharan small-scale farmers in accessing farming information through mobile phones: a systematic literature review. *Electr. J. Inform. Syst. in Dev. Countries* 84:e12034 10.1002/isd2.12034

[B26] Mohammed I. Abarshi M. Muli B. Hillocks R. Maruthi M. (2012). The symptom and genetic diversity of cassava brown streak viruses infecting cassava in East Africa. *Adv. Virol.* 2012:795697. 10.1155/2012/795697 22454639 PMC3290829

[B27] Monger W. Seal S. Isaac A. Foster G. (2001). Molecular characterization of the Cassava brown streak virus coat protein. *Plant Pathol.* 50 527–534. 10.1046/j.1365-3059.2001.00589.x

[B28] Ndunguru J. Legg J. P. Aveling T. A. S. Thompson G. Fauquet C. M. (2005). Molecular biodiversity of cassava begomoviruses in Tanzania: evolution of cassava geminiviruses in Africa and evidence for East Africa being a canter of diversity of cassava geminiviruses. *Virol. J.* 2:21. 10.1186/1743-422X-2-21 PMC1079959 15784145

[B29] Nichols J. (1950). The brown streak disease of cassava: distribution, climatic effects and diagnostic symptoms. *East African Agric. J.* 15 154–160. 10.1080/03670074.1950.11664727

[B30] Onzo A. Hanna R. Sabelis M. W. (2005). Biological control of cassava green mites in Africa: impact of the predatory mite Typhlodromalus aripo. *Entomol. Berichten.* 65 2–7.

[B31] Owor B. Legg J. Obonyo R. Okao-Okuja G. Kyamanywa S. Ogenga-Latigo M. W. (2004a). Field studies of cross protection with cassava in Uganda. *J. Phytopathol.* 152 243–249. 10.1111/j.1439-0434.2004.00837.x

[B32] Owor B. Legg J. Okao-Okuja G. Obonyo R. Ogenga-Latigo M. (2004b). The effect of cassava mosaic geminiviruses on symptom severity, growth and root yield of a cassava mosaic virus disease-susceptible cultivar in Uganda. *Ann. Appl. Biol.* 145 331–337. 10.1111/j.1744-7348.2004.tb00390.x

[B33] Pariona A. (2019). *Most Important Staple Foods in the world.* Available online at: https://www.worldatlas.com/articles/most-important-staple-foods-in-the-world.html (accessed on 25 May 2020).

[B34] Pethybridge S. Nelson S. (2015). Leaf doctor: a new portable application for quantifying plant disease severity. *Plant Dis.* 99 1310–1316. 10.1094/PDIS-03-15-0319-RE 30690990

[B35] Prasad S. Peddoju S. K. Ghosh D. (2016). Multi-resolution mobile vision system for plant leaf disease diagnosis. *Signal Image Video Process.* 10 379–388. 10.1007/s11760-015-0751-y

[B36] Qiang C. Kuek S. Dymond A. Esselaar S. (2012). *Mobile Applications for Agriculture and Rural Development.* Washington, DC: World Bank.

[B37] Qin F. Liu D. Sun B. Ruan L. Ma Z. Wang H. (2016). Identification of alfalfa leaf diseases using image recognition technology. *PLoS One* 11:e0168274. 10.1371/journal.pone.0168274 27977767 PMC5158033

[B38] Ramcharan A. Baranowski K. McCloskey P. Ahmed B. Legg J. Hughes D. (2017). Deep learning for image-based cassava disease detection. *Front. Plant Sci.* 8:1852. 10.3389/fpls.2017.01852 29163582 PMC5663696

[B39] Ramcharan A. McCloskey P. Baranowski K. Mbilinyi N. Mrisho L. Ndalahwa M. (2019). A mobile-based deep learning model for cassava disease diagnosis. *Front. Plants Sci.* 10:272. 10.3389/fpls.2019.00272 30949185 PMC6436463

[B40] Shirima R. Legg J. Maeda D. Tumwegamire S. Mkamilo G. Mtunda K. (2020). Genotype by environment cultivar evaluation for cassava brown streak disease resistance in Tanzania. *Virus Res.* 286:198017. 10.1016/j.virusres.2020.198017 32461191 PMC7450270

[B41] Shirima R. Maeda D. Kanju E. Tumwegamire S. Ceasar G. Mushi E. (2019). Assessing the degeneration of cassava under high virus inoculum conditions in Coastal Tanzania. *Plant Dis.* 103 2652–2664. 10.1094/PDIS-05-18-0750-RE 31322490 PMC7779971

[B42] Sladojevic S. Arsenovic M. Anderla A. Culibrk D. Stefanovic D. (2016). Deep neural networks based recognition of plant diseases by leaf image classification. *Comput. Intell. Neurosci.* 2016:3289801. 10.1155/2016/3289801 27418923 PMC4934169

[B43] Sseruwagi P. Sserubombwe W. Legg J. Ndunguru J. Thresh J. M. (2004). Methods of surveying the incidence and severity of cassava mosaic disease and whitefly vector populations on cassava in Africa: a review. *Virus Res.* 100 129–142. 10.1016/j.virusres.2003.12.021 15036844

[B44] StatCounter Global Stats (2020). *Mobile Operating System Market Share Africa.* Available online at: https://gs.statcounter.com/os-market-share/mobile/africa (accessed on 18 May 2020).

[B45] Thresh J. Cooter J. (2005). Strategies for controlling cassava mosaic virus disease in Africa. *Plant Pathol.* 54 587–614. 10.1111/j.1365-3059.2005.01282.x

[B46] Thresh J. Otim-Nape G. Jennings D. (1994). Exploiting resistance to African cassava mosaic virus. *Aspects Appl. Biol.* 39 51–60.

[B47] Tsan M. Totapally S. Hailu M. Addom B. K. (2019). *The Digitalisation of African Agriculture Report 2018-2019.* Chicago, IL: CTA.

[B48] Tuhaise J. Quinn J. A. Mwebaze E. (2014). “Pixel classification methods for automatic symptom measurement of cassava brown streak disease,” in *Proceding of the 1st International Conference on the Use of Mobile ICT in Africa*, Stellenbosch.

