# Supplementary material for: Accuracy of a Smartphone-Based Object Detection Model, PlantVillage Nuru, in Identifying the Foliar Symptoms of the Viral Diseases of Cassava–CMD and CBSD
Source: Front Plant Sci. 2020 Dec 18;11:590889. doi: 10.3389/fpls.2020.590889 (PMC7775399; doi:10.3389/fpls.2020.590889)
Supplement: Supplementary file 1 [file Data_Sheet_1.PDF]

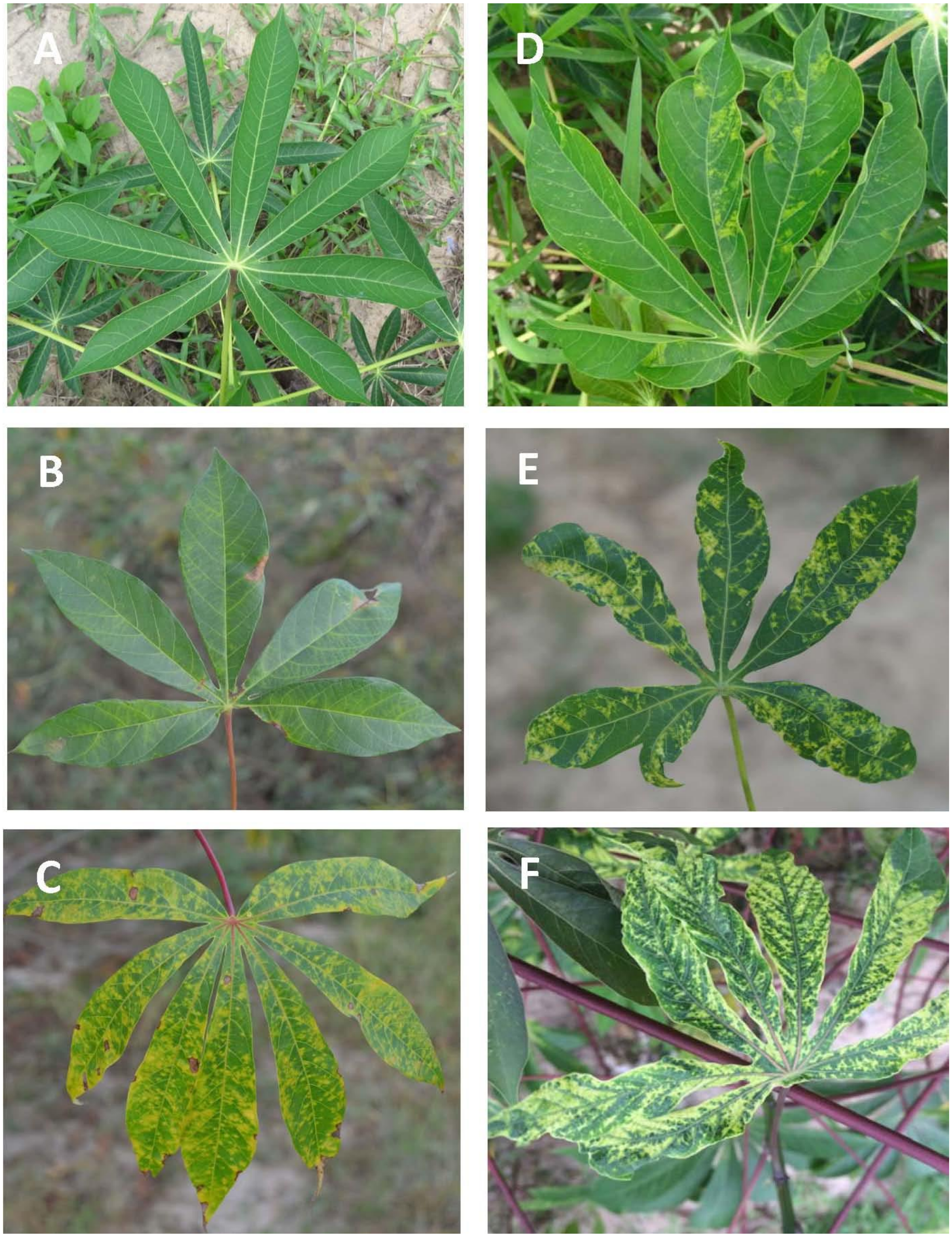

Figure 1: Cassava leaf from a healthy plant (A) and CBSD-infected plant showing mild (B) and intermediate (C) leaf symptoms as well as CMD-infected plants showing mild (D), intermediate (E) and severe (F) symptoms.

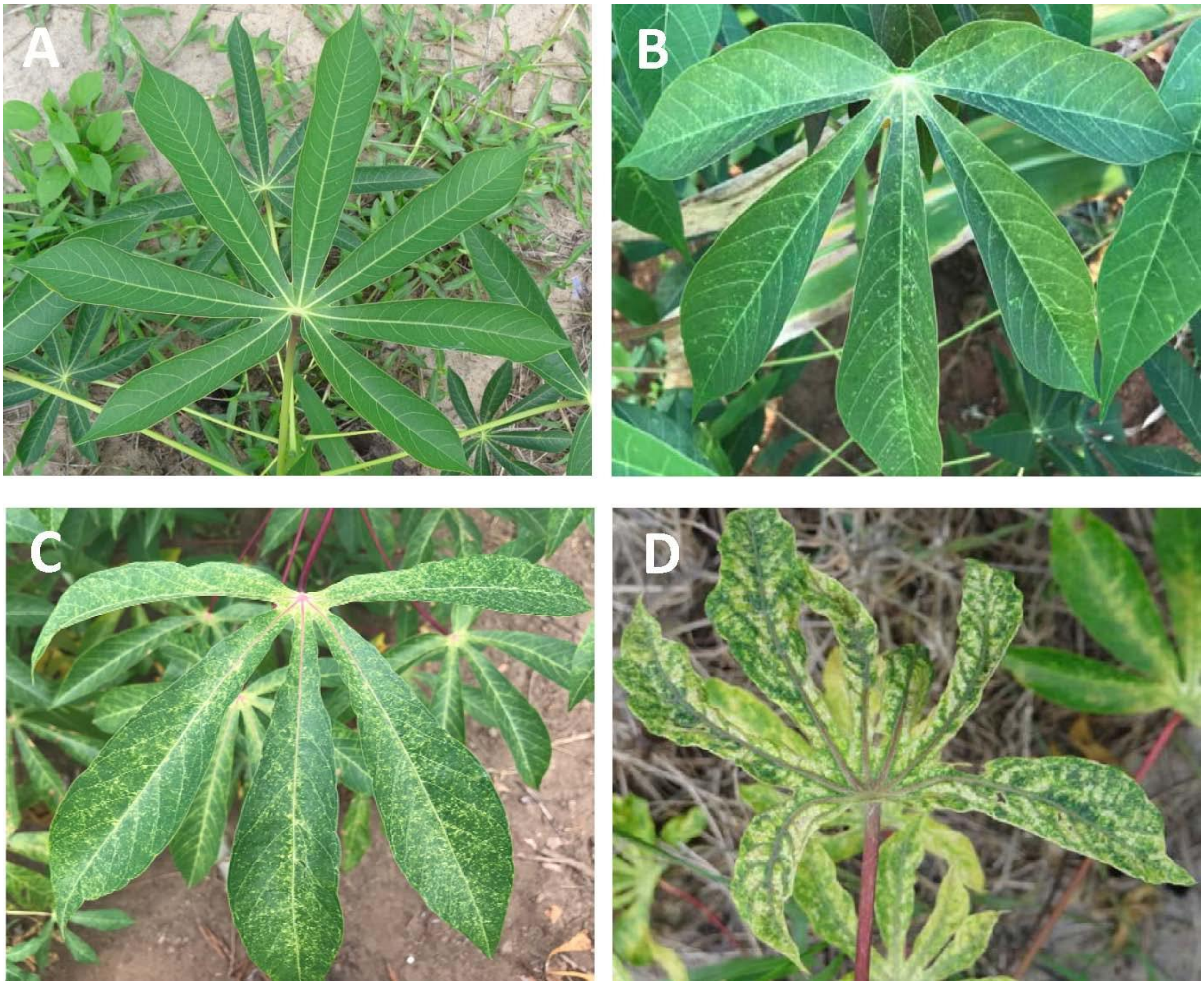

Figure 2: Cassava leaf from a healthy plant (A) and CGM-infested plant showing mild (B), intermediate (C) and severe (D) leaf symptoms.
