# Supplementary material for: Accuracy of a Smartphone-Based Object Detection Model, PlantVillage Nuru, in Identifying the Foliar Symptoms of the Viral Diseases of Cassava–CMD and CBSD
Source: Front Plant Sci. 2020 Dec 18;11:590889. doi: 10.3389/fpls.2020.590889 (PMC7775399; doi:10.3389/fpls.2020.590889)
Supplement: Supplementary file 2 [file Data_Sheet_2.PDF]

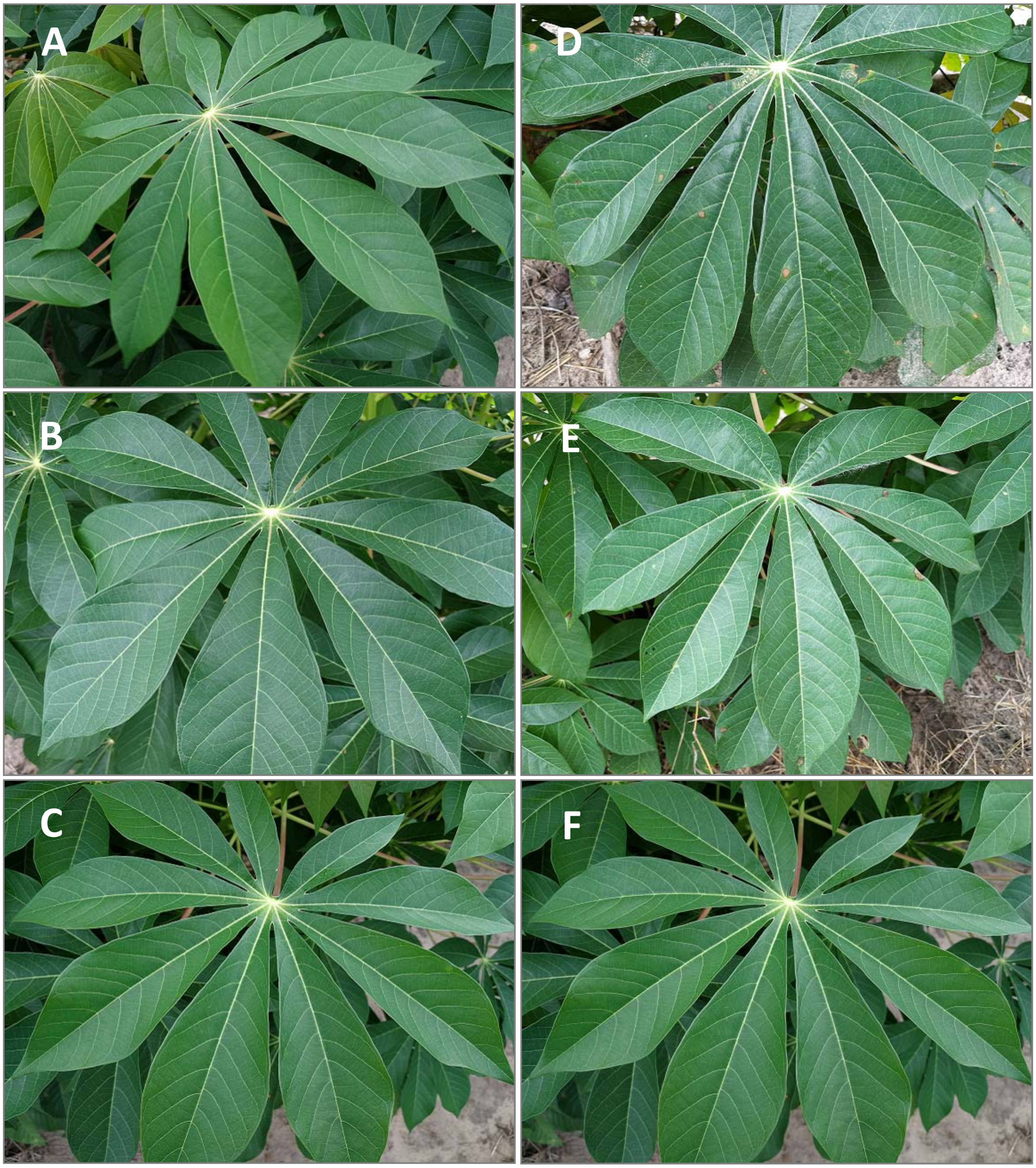

Figure 1: Example of cassava leaves from asymptomatic plant showing healthy leaves that were used to assess the effect of using multiple leaves for improving the diagnostic capability of PlantVillage Nuru. A - C represent the first, second and third upper leaves; D - F represent the first, second and third lower leaves.

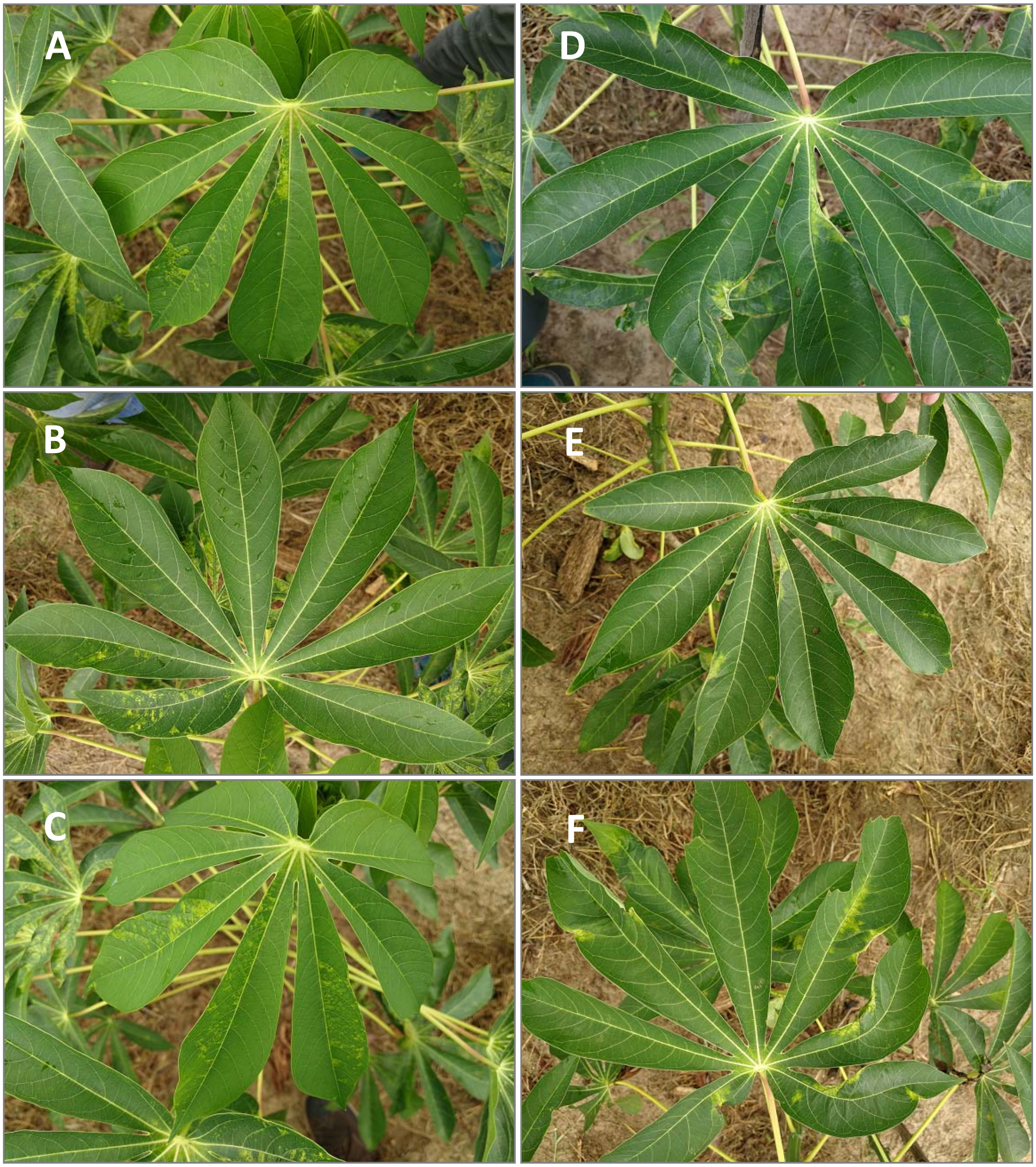

Figure 2: Examples of cassava leaves from symptomatic plant, with mild symptoms of Cassava mosaic disease (CMD), that were used to assess the effect of using multiple leaves for improving the diagnostic capability of PlantVillage Nuru. A - C represent the leaves from the second and third upper leaves; D - F represent the first, second and third lower leaves.

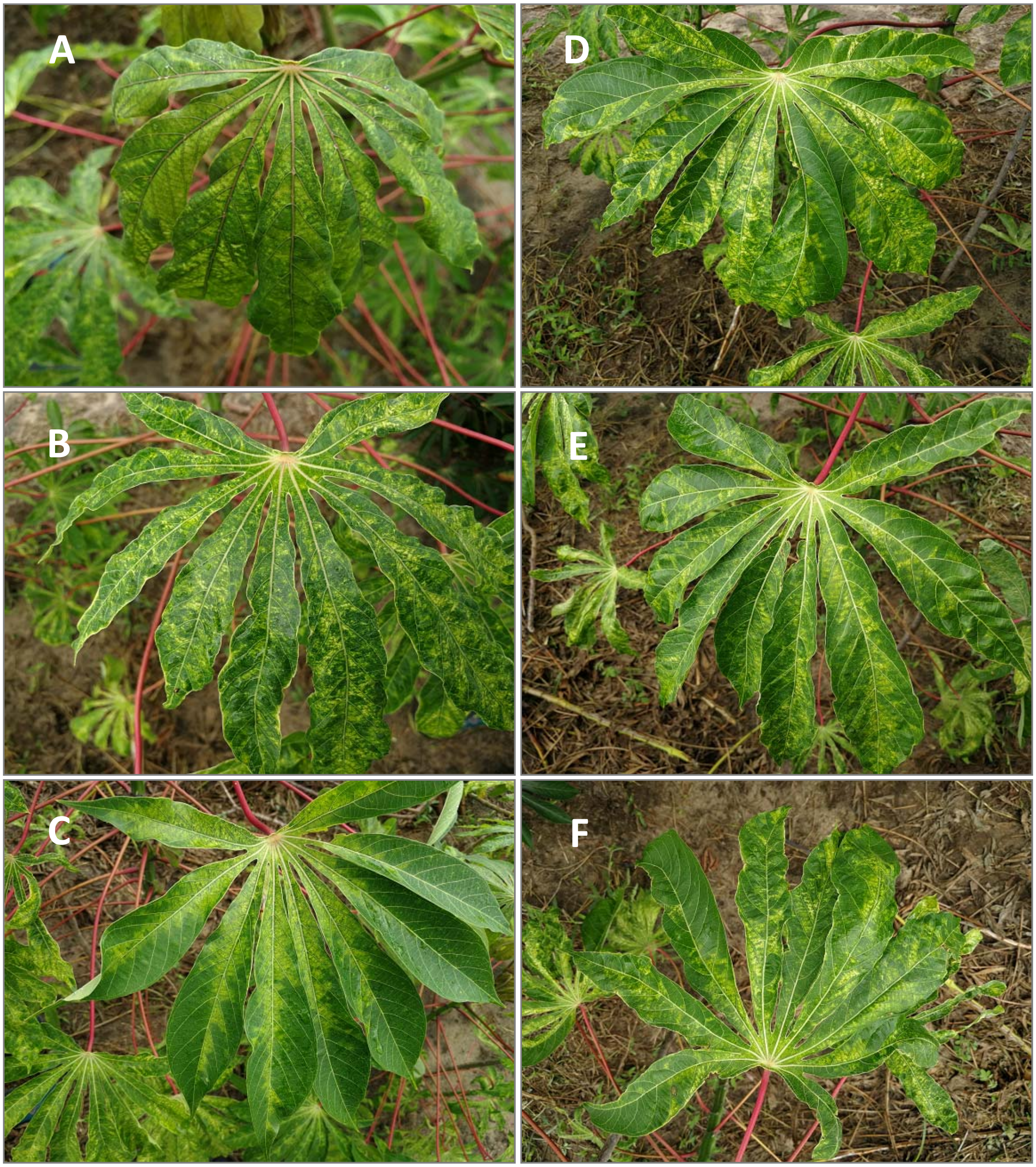

Figure 3: Example of cassava leaves from symptomatic plant, with moderate symptoms of Cassava mosaic disease (CMD), that were used to assess the effect of using multiple leaves for improving the diagnostic capability of PlantVillage Nuru. A - C represent the leaves from the second and third upper leaves; D - F represent the first, second and third lower leaves.

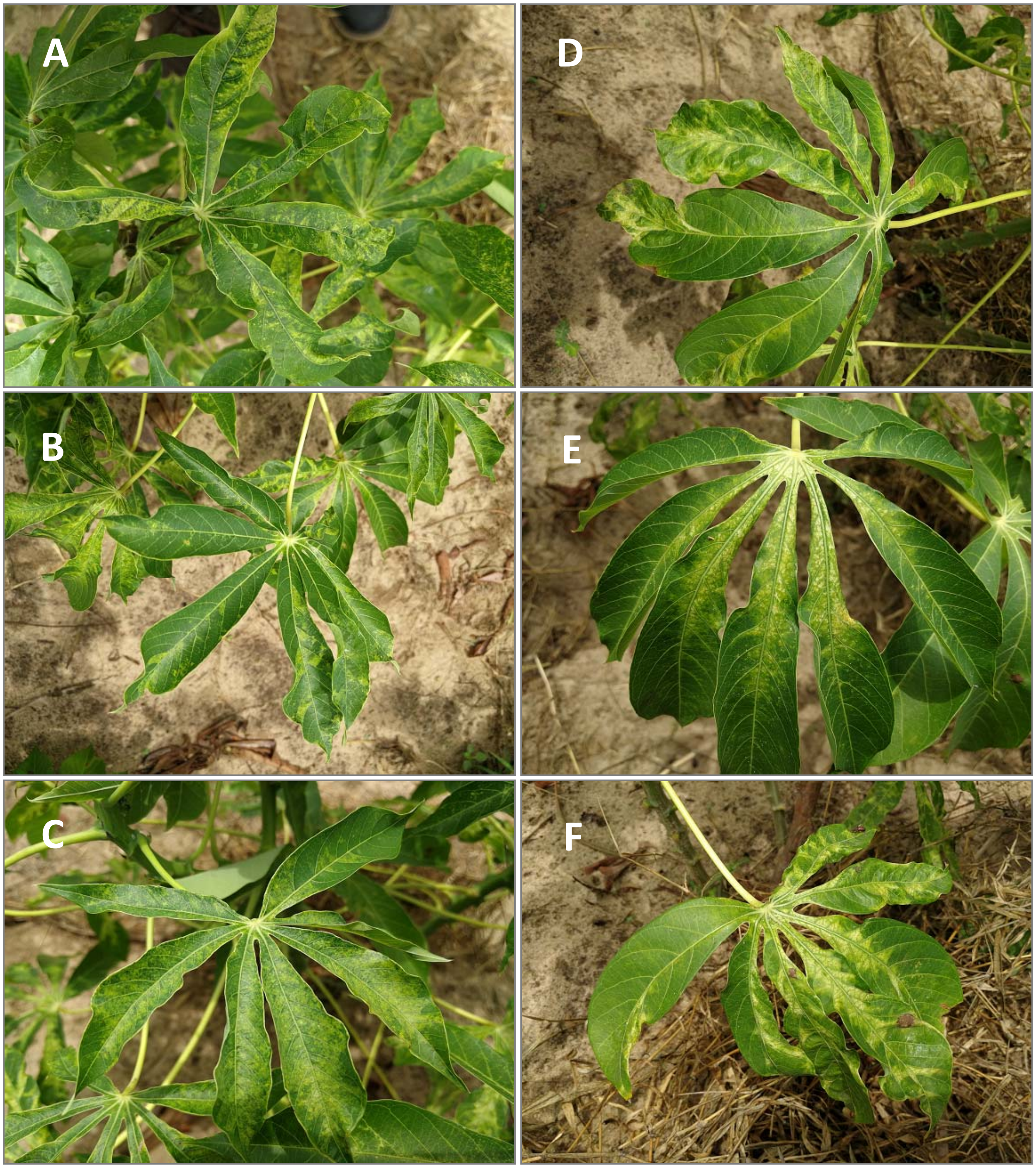

Figure 4: Example of cassava leaves from symptomatic plant, with unclear symptoms of Cassava mosaic disease (CMD), that were used to assess the effect of using multiple leaves for improving the diagnostic capability of PlantVillage Nuru. A - C represent the leaves from the second and third upper leaves; D - F represent the first, second and third lower leaves.

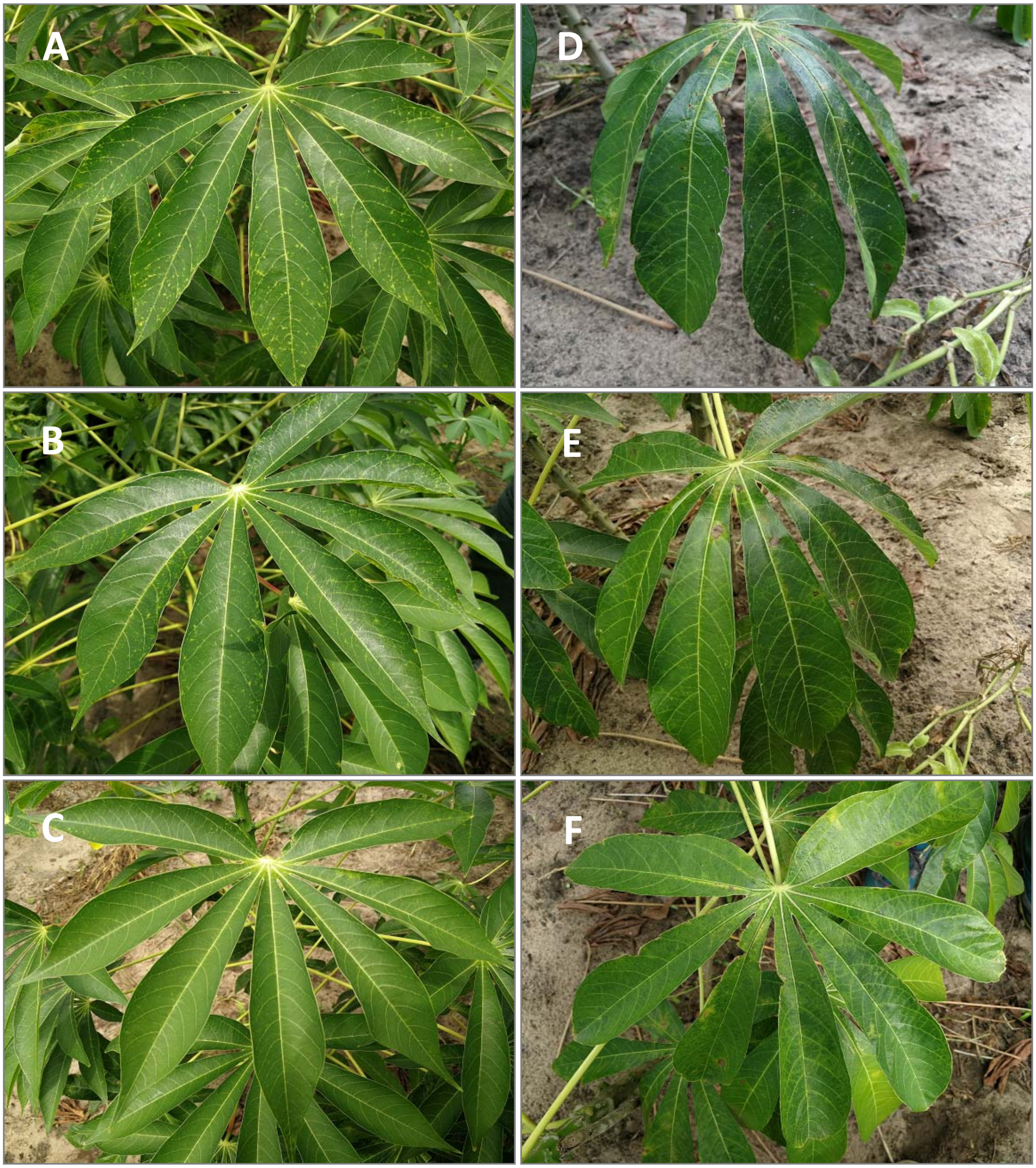

Figure 5: Examples of cassava leaves from symptomatic plant, with mild symptoms of Cassava brown streak disease (CBSD), that were used to assess the effect of using multiple leaves for improving the diagnostic capability of PlantVillage Nuru. A - C represent the leaves from the second and third upper leaves; D - F represent the first, second and third lower leaves.

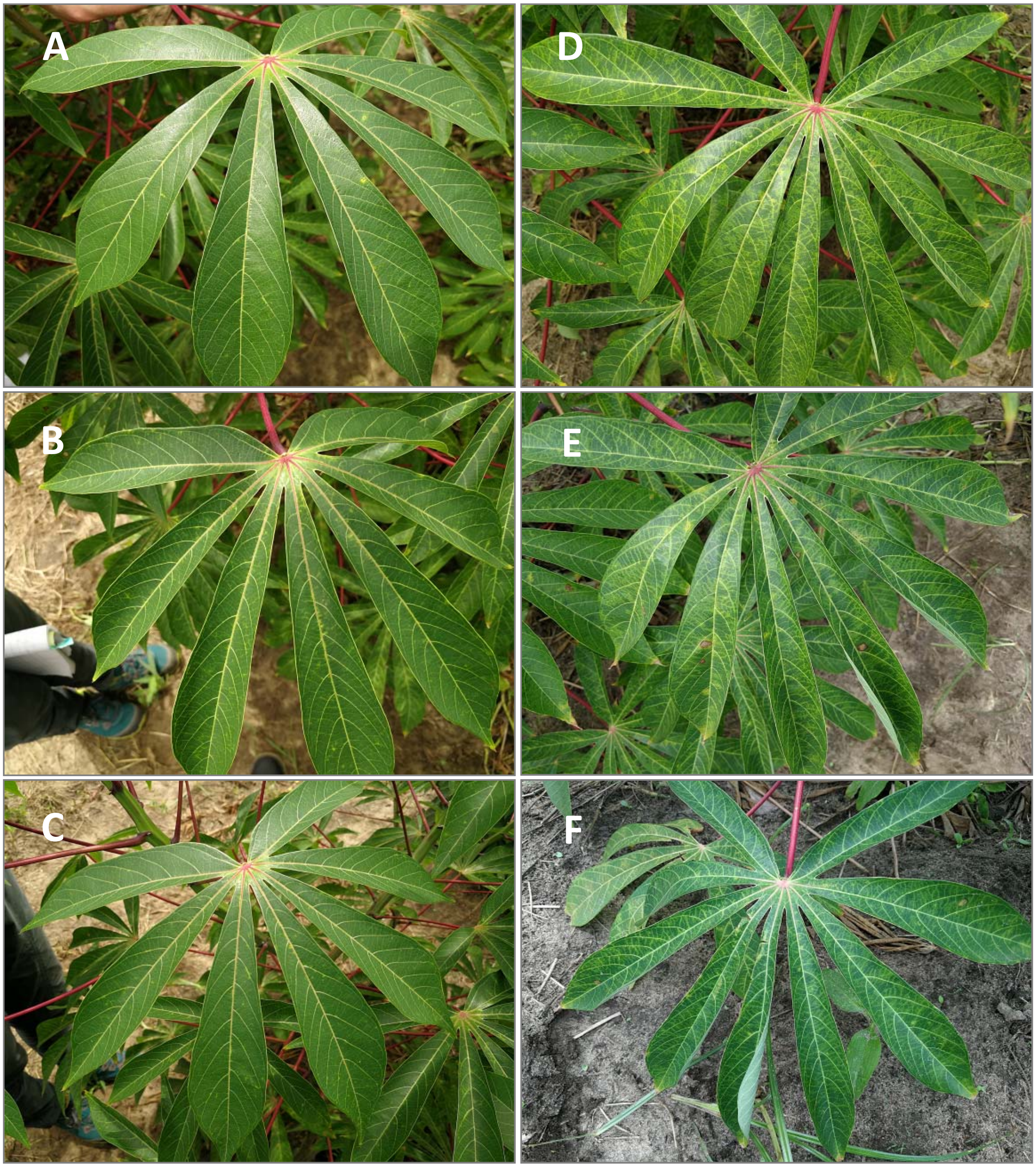

Figure 6: Examples of cassava leaves from symptomatic plant, with moderate symptoms of Cassava brown streak disease (CBSD), that were used to assess the effect of using multiple leaves for improving the diagnostic capability of PlantVillage Nuru. A - C represent the leaves from the second and third upper leaves; D - F represent the first, second and third lower leaves.

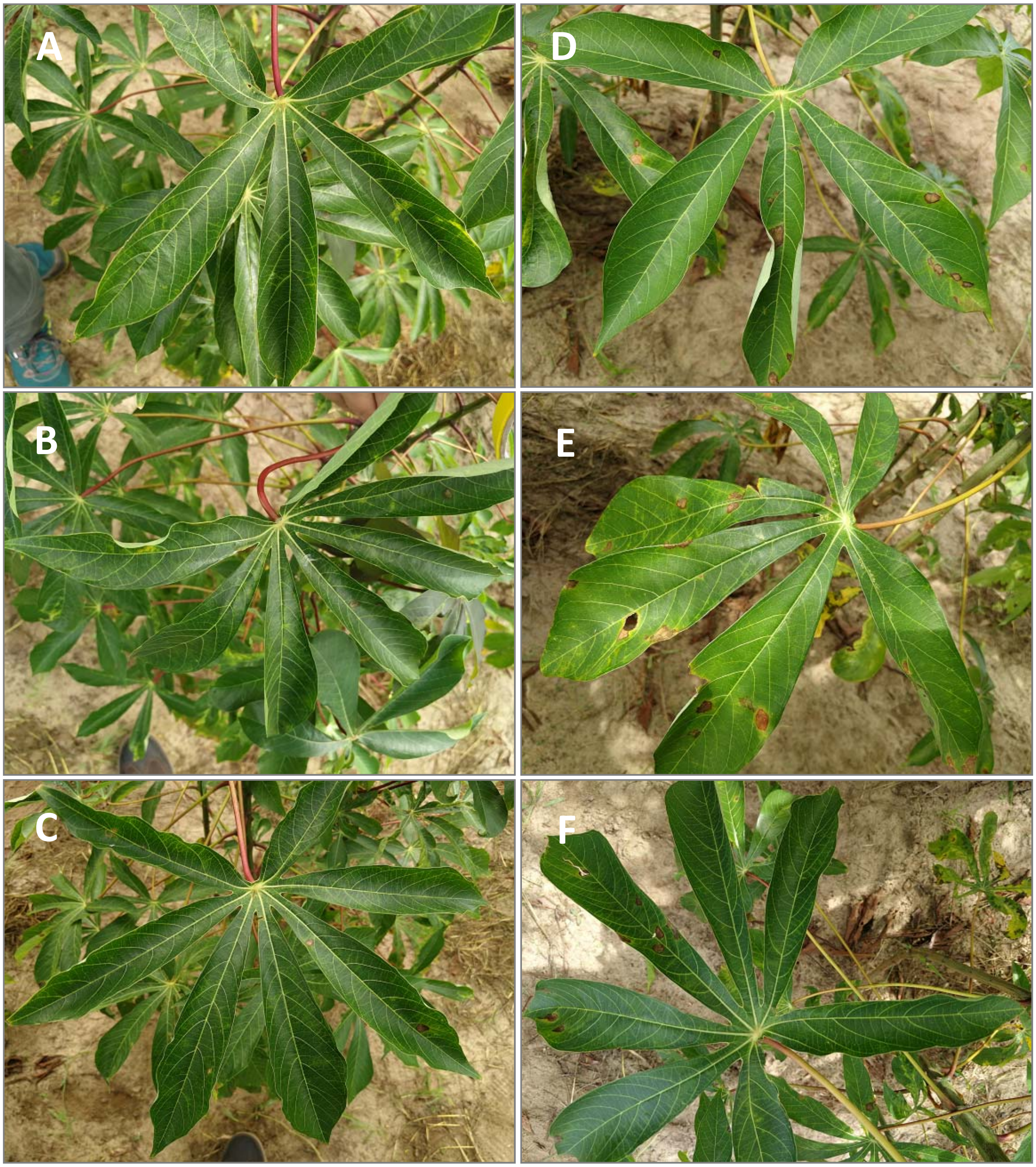

Figure 7: Examples of cassava leaves from symptomatic plant, with unclear symptoms of cassava brown streak disease (CBSD), that were used to assess the effect of using multiple leaves for improving the diagnostic capability of PlantVillage Nuru. A - C represent the leaves from the second and third upper leaves; D - F represent the first, second and third lower leaves.

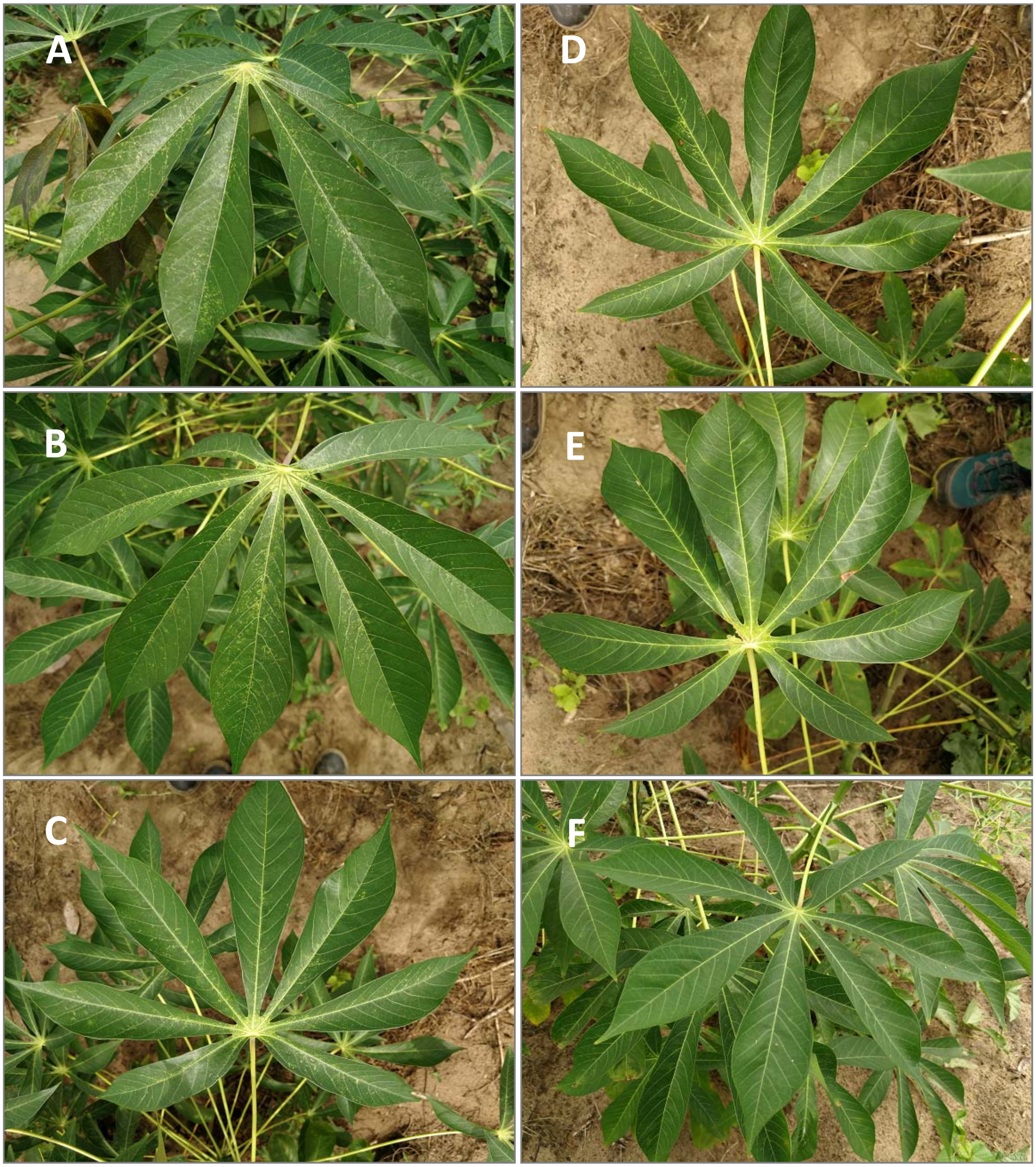

Figure 8: Example of cassava leaves from symptomatic plant, with mild symptoms of damage caused by cassava green mites (CGM-damage), that were used to assess the effect of using multiple leaves for improving the diagnostic capability of PlantVillage Nuru. A - C represent the leaves from the second and third upper leaves; D - F represent the first, second and third lower leaves.

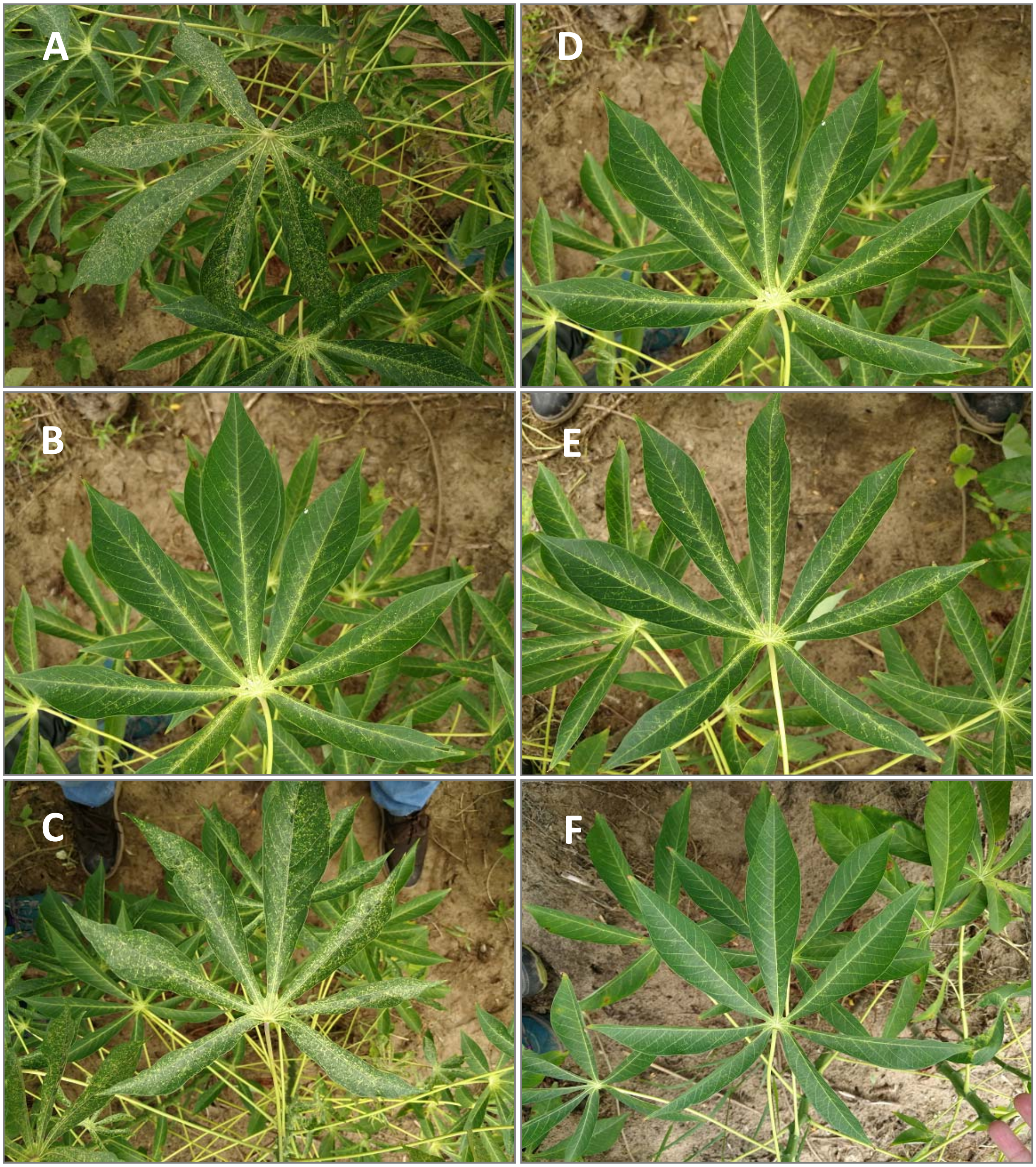

Figure 9: Example of cassava leaves from symptomatic plant, with moderate symptoms of damage caused by cassava green mites (CGM-damage), that were used to assess the effect of using multiple leaves for improving the diagnostic capability of PlantVillage Nuru. A - C represent the leaves from the second and third upper leaves; D - F represent the first, second and third lower leaves.

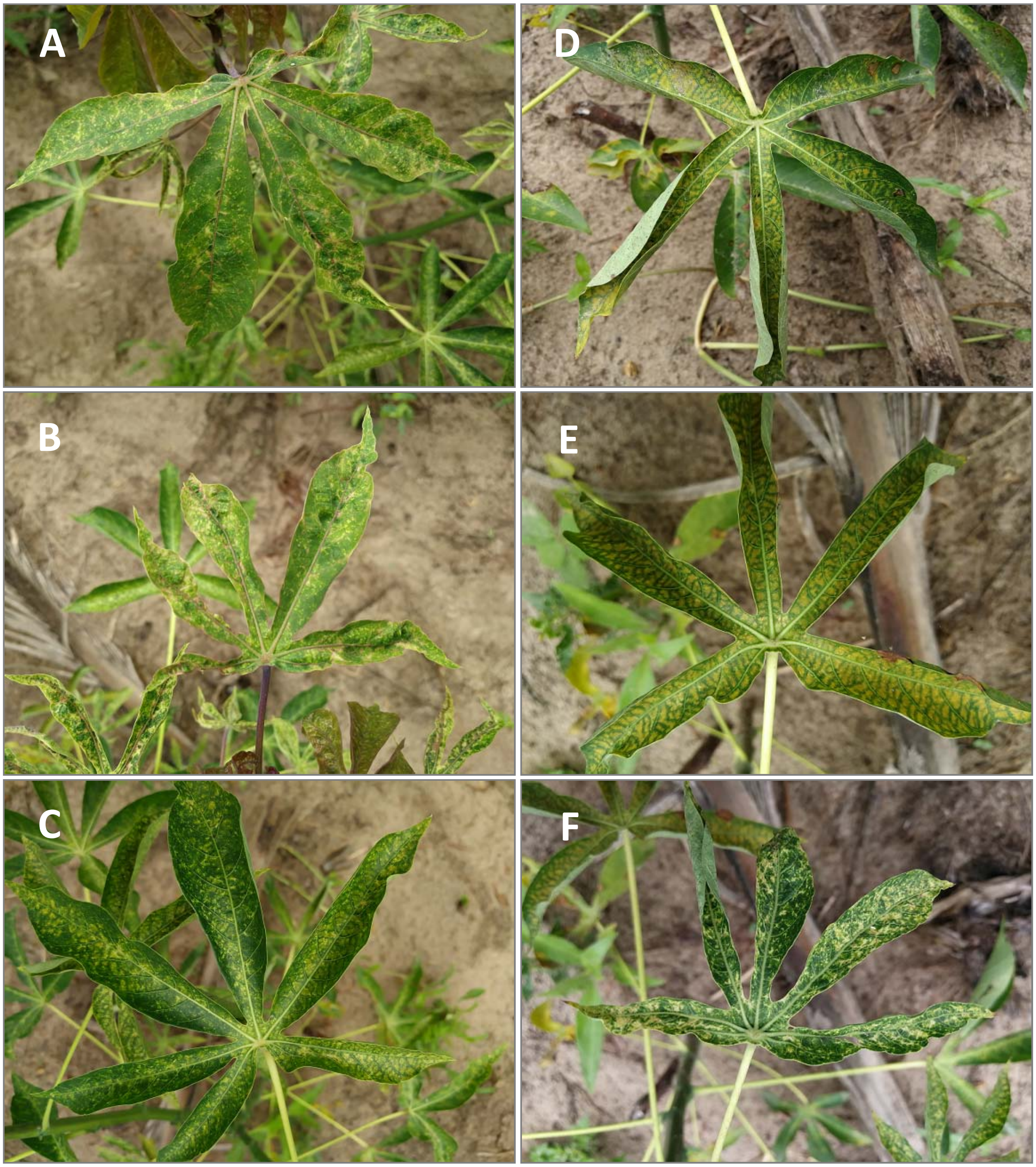

Figure 10: Example of cassava leaves from symptomatic plant, with unclear symptoms of damage caused by cassava green mites (CGM-damage), that were used to assess the effect of using multiple leaves for improving the diagnostic capability of PlantVillage Nuru. A - C represent the leaves from the second and third upper leaves; D - F represent the first, second and third lower leaves.
