# Supplementary material for: Accuracy of a Smartphone-Based Object Detection Model, PlantVillage Nuru, in Identifying the Foliar Symptoms of the Viral Diseases of Cassava–CMD and CBSD
Source: Front Plant Sci. 2020 Dec 18;11:590889. doi: 10.3389/fpls.2020.590889 (PMC7775399; doi:10.3389/fpls.2020.590889)
Supplement: Supplementary file 3 [file Data_Sheet_3.PDF]

Development of the cassava symptom recognition assessment tool (CaSRAT)

The cassava symptom recognition assessment tool consist of a scoring system for 170 images of cassava leaves which are asymptomatic and symptomatic for CMD and CBSD as well as CGM-damage. The system was developed by 10 cassava experts, from the International Institute of Tropical Agriculture - IITA, Tanzania, who have been working on cassava pests and diseases for 2 to over 10 years. The experts individually scored 170 images of the cassava leaves based on the symptoms they recognized on the leaves using the scoring key illustrated in Table 1. Brown leaf spot (BLS) and Fungal-like infection (FLI) were also added in the conditions that could be identified, even though these conditions were not present in the 170 images, because these conditions are commonly found in cassava plants and we wanted to see if they were confused with symptoms of CMD, CBSD and CGM-damage.

The score obtained from each of cassava expert were compared to determine images that were given the same scores by the experts and those that were scored differently by more than three experts. Images whose scores were not consistent were identified and discussed to determine the correct consensus diagnosis. The scores representing the consensus expert diagnosis for all the 170 images were considered as the expert scores and were used generate a comparison matrix which was used to compare scores entered by the individuals being evaluated. The expert scores were used to calculate the individual's accuracy of symptom recognition, i.e. the percentage of images that were correctly identified by the individual being assessed, based on the expert's score. The comparison matrix also enabled identification of the images that were incorrectly diagnosed and analysing the conditions which were misdiagnosed to inform on diagnosis given on the images that were misdiagnosed, example if the individual being assessed misdiagnosed CMD and CGM-damage or FLI as CBSD.

Table 1: Numerical codes to identify the conditions of the leaf images used for the development of the cassava symptom recognition assessment tool.

Condition	Abbreviation	Score
Cassava mosaic disease	CMD	1
Cassava brown streak disease	CBSD	2
Cassava green mites	Mites	3
Brown leaf spots	BLS	4
Fungal-like infection	FLI	5
Healthy	-	6
Other	-	7
Not sure	-	8
