# Supplementary material for: Accuracy of a Smartphone-Based Object Detection Model, PlantVillage Nuru, in Identifying the Foliar Symptoms of the Viral Diseases of Cassava–CMD and CBSD
Source: Front Plant Sci. 2020 Dec 18;11:590889. doi: 10.3389/fpls.2020.590889 (PMC7775399; doi:10.3389/fpls.2020.590889)
Supplement: Supplementary file 4 [file Data_Sheet_4.PDF]

SUPPLEMENTRAY E

Table E1: Analysis of images that were misdiagnosed by farmers, showing the total number of images that were misdiagnosed from the 1700 images that were analysed.

Expert diagnosis	Number of misdiagnosed leaves	Diagnosis given by untrained farmers					
		CMD	CBSD	Mites	Healthy	Other	% Misdiagnoses
CMD	108	-	4%	2%	6%	1%	13%
CBSD	349	9%	-	3%	24%	7%	42%
CGM	311	6%	14%	-	14%	3%	37%
Co-infection	65	1%	2%	0%	3%	1%	8%
Total	833						100%

Expert diagnosis	Number of misdiagnosed leaves	Diagnosis given by trained farmers					
		CMD	CBSD	Mites	Healthy	Other	% Misdiagnoses
CMD	76	-	4%	3%	2%	2%	11%
CBSD	270	9%	-	12%	9%	8%	38%
CGM	280	9%	20%	-	8%	3%	39%
Co-infection	86	2%	2%	1%	3%	4%	12%
Total	712						100%

Table E2: Analysis of images that were misdiagnosed by agricultural extension officers, showing the total number of images that were misdiagnosed from the 1700 images that were analysed.

Expert diagnosis	Number of misdiagnosed leaves	Diagnosis given by untrained extension officers					
		CMD	CBSD	Mites	Healthy	Other	% Misdiagnoses
CMD	88	-	9.8%	1.7%	0.5%	0.6%	13%
CBSD	235	22.1%	-	5.5%	5.6%	1.7%	35%
CGM	308	16.7%	23.0%	-	4.4%	1.2%	45%
Co-infection	53	2.2%	2.8%	1.4%	0.8%	0.4%	8%
Total	684						100%

Expert diagnosis	Number of misdiagnosed leaves	Diagnosis given by trained extension officers					
		CMD	CBSD	Mites	Healthy	Other	% Misdiagnoses
CMD	50	-	9.2%	1.0%	1.3%	0.2%	12%
CBSD	127	11.3%	-	10.4%	8.5%	0.0%	30%
CGM	192	6.3%	27.5%	-	10.0%	0.2%	44%
Co-infection	62	1.7%	5.0%	6.2%	0.4%	0.6%	14%
Total	430						100%

Table E3: Analysis of images that were misdiagnosed by researchers, showing the total number of images that were misdiagnosed from the 1700 images that were analysed.

Expert diagnosis	Number of misdiagnosed leaves	Diagnosis given by untrained researchers					
		CMD	CBSD	Mites	Healthy	Other	% Misdiagnoses
CMD	122	-	6.1%	2.2%	0.8%	3.6%	13%
CBSD	399	11.1%	-	9.6%	7.0%	13.5%	41%
CGM	374	12.0%	11.7%	-	5.7%	9.2%	39%
Co-infection	73	1.7%	2.7%	0.5%	1.0%	1.6%	8%
Total	968						100%

Expert diagnosis	Number of misdiagnosed leaves	Diagnosis given by trained researchers					
		CMD	CBSD	Mites	Healthy	Other	% Misdiagnoses
CMD	20	-	6.4%	2.3%	0.4%	0.0%	9%
CBSD	46	12.4%	-	2.3%	0.4%	5.6%	21%
CGM	75	11.6%	12.4%	-	6.7%	3.0%	34%
Co-infection	89	6.7%	15.0%	10.9%	2.6%	1.5%	37%
Total	230						100%

Table E4: Analysis of images that were misdiagnosed by agricultural extension officers

Expert diagnosis	Number of misdiagnosed leaves	Diagnosis given by 33 agricultural extension officers before training (from a total of 5610 images that were analyzed)					
		CMD	CBSD	Mites	Healthy	Other	% Misdiagnoses
CMD	103	-	6.0%	4.0%	1.0%	0.0%	11%
CBSD	427	19.0%	-	22.0%	6.0%	0.0%	47%
CGM	300	12.0%	16.0%	-	5.0%	0.0%	33%
Co-infection	78	2.0%	2.0%	3.0%	2.0%	0.0%	9%
Total	908						100%

Expert diagnosis	Number of misdiagnosed leaves	Diagnosis given by 10 agricultural extension officers after training (from a total of 1700 images that were analyzed)					
		CMD	CBSD	Mites	Healthy	Other	% Misdiagnoses
CMD	74	-	9.0%	3.0%	1.0%	0.0%	13%
CBSD	254	14.0%	-	27.0%	4.0%	0.0%	45%
CGM	162	8.0%	16.0%	-	4.0%	0.0%	28%
Co-infection	72	1.6%	3.6%	7.3%	1.0%	0.0%	14%
Total	562						100%

Expert diagnosis	Number of misdiagnosed leaves	Diagnosis given by 10 agricultural extension officers after training and usage of PlantVillage Nuru for a period of two weeks (from a total of 1700 images that were analyzed)					
		CMD	CBSD	Mites	Healthy	Other	% Misdiagnoses
CMD	53	-	9.3%	1.9%	0.0%	0.0%	11%
CBSD	187	16.4%	-	18.9%	4.5%	0.0%	40%
CGM	172	12.3%	20.0%	-	4.2%	0.0%	37%
Co-infection	59	3.2%	2.5%	6.2%	0.6%	0.0%	13%
Total	471						100%
